# Activity-Dependent Changes in Axonal Action Potential Latency Coordinated with Synaptic Potentiation in Individual Hippocampal Neurons

**DOI:** 10.1523/ENEURO.0114-26.2026

**Published:** 2026-08-19

**Authors:** Yoshihiko Yamazaki, Hiroki Fujiwara

**Affiliations:** Department of Physiology, Yamagata University School of Medicine, Yamagata 990-9585, Japan

**Keywords:** action potential, hippocampus, long-term potentiation

## Abstract

Neural plasticity enables the nervous system to adapt its structure and function in response to experience. Although synaptic plasticity is a central cellular mechanism underlying learning and memory, action potential propagation along axons is also subject to plastic regulation and critically shapes neural computation. However, how these distinct forms of plasticity are coordinated within individual neurons remains poorly understood. Here, we simultaneously monitored synaptic responses and antidromically evoked action potentials in hippocampal CA1 pyramidal neurons from male rats using whole-cell recordings. High-frequency stimulation reliably induced long-term potentiation (LTP) and was accompanied by a delayed yet transient reduction in antidromic action potential latency. The magnitude of latency shortening correlated with the degree of synaptic potentiation across multiple post-high-frequency stimulation time windows, including a late phase during which synaptic responses remained persistently elevated. Blocking the induction of LTP by intracellular Ca^2+^ chelation or NMDA receptor antagonism abolished latency shortening, indicating a dependence on LTP-related signaling pathways. Notably, the temporal profile of latency modulation depended on the site of axonal stimulation: latency shortening emerged earlier at proximal sites and was delayed at more distal sites, suggesting the propagation or accumulation of plasticity-related signals along an axon. Dual-site axonal stimulation within single neurons further demonstrated that action potential latency changes differed across axonal locations. Together, these findings demonstrate that synaptic potentiation is accompanied by spatially and temporally organized latency changes, revealing an additional layer of activity-dependent plasticity within individual neurons.

## Significance Statement

Neuronal communication depends not only on the strength of synaptic transmission but also on the timing of action potential generation and arrival. Although synaptic plasticity has been studied extensively, it remains largely unknown whether action potential timing is dynamically regulated in association with synaptic plasticity. Here, we show that long-term synaptic potentiation in hippocampal neurons is accompanied by delayed, transient, and spatially organized changes in axonal action potential latency. These latency changes require NMDA receptor-dependent signaling and vary along axonal locations. Our findings reveal that synaptic plasticity can coordinate changes in synaptic strength with the timing of action potentials within individual neurons.

## Introduction

The nervous system adapts structurally and functionally in response to external stimuli, internal states, and experience. This adaptive capacity, commonly referred to as neural plasticity (von Bernhardi et al., 2017), enables the lifelong modification of neural structure, connectivity, and activity and is a central focus of research spanning molecular, cellular, systems, and cognitive neuroscience. Over the past few decades, substantial progress has been made in elucidating the mechanisms and functional consequences of neural plasticity across multiple levels of organization in the nervous system (Gazerani, 2025). Among the most studied forms of functional plasticity is synaptic plasticity, which is widely regarded as the cellular basis of learning and memory (Bliss and Lomo, 1973; Takeuchi et al., 2014; Abraham et al., 2024); however, neural plasticity is increasingly acknowledged as a multicomponent process involving coordinated changes in neuronal and non-neuronal elements. Activity-dependent modifications of intrinsic excitability, glial function, dendritic structure, and extracellular matrix organization have been implicated in experience-dependent circuit remodeling (Mozzachiodi and Byrne, 2010; Kol and Goshen, 2021). In this broader context, it is accepted that action potential conduction along axons is subject to dynamic, activity-dependent regulation (Debanne et al., 2011; Ford et al., 2015; Etxeberria et al., 2016). Recent studies have highlighted the contribution of adaptive myelination and oligodendrocyte-dependent mechanisms to the activity-dependent regulation of conduction properties, indicating that axonal conduction is itself a dynamic component of neural circuit plasticity (Wake et al., 2011; Fields, 2015; Knowles et al., 2022). This phenomenon, often referred to as axonal conduction plasticity, has been demonstrated in myelinated and unmyelinated fibers. In myelinated fibers, axonal conduction exhibits functional plasticity, e.g., in the adult rodent brain, modulation of oligodendrocyte NKCC1 activity contributes to the activity-dependent regulation of axonal conduction in mature myelinated axons (Yamazaki et al., 2021). In unmyelinated fibers, activity-dependent changes in axonal conduction have also been reported, e.g., in hippocampal mossy fibers (Chida et al., 2015), indicating that conduction plasticity occurs in distinct structural classes of axons.

Given that information processing in the nervous system relies on synaptic transmission and action potential propagation, it is plausible that plasticity in both mechanisms is required for the emergence of complex brain functions. Indeed, even highly simplified models of learning and memory cannot be adequately explained by considering either synaptic transmission or axonal conduction in isolation. Therefore, it is essential not only to elucidate the individual mechanisms underlying each form of plasticity but also to understand how these distinct processes converge and interact within neural circuits. Nevertheless, the dynamic interplay between different types of functional plasticity remains largely unexplored. In particular, the temporal and spatial organization of activity-dependent changes in axonal conduction within individual neurons has not been examined systematically.

Enhancing axonal conduction can influence synaptic integration by synchronizing the arrival of multiple action potentials and thereby promoting the temporal summation of excitatory inputs (Bucher and Goaillard, 2011; Debanne et al., 2011). Such an enhancement of axonal conduction has also been shown to facilitate the induction of long-term potentiation (LTP) in postsynaptic target neurons (Yamazaki et al., 2019). These findings suggest that axonal conduction is not merely a passive conduit for signal transmission but is an active contributor to synaptic processing. Building on this concept, it is conceivable that the induction of synaptic plasticity may, in turn, be accompanied by adaptive changes in the axonal conduction properties of the same neurons. Investigating this relationship may help clarify how distinct forms of neural plasticity are coordinated within single neurons and contribute to the regulation of brain function through integrated plastic changes. In this study, we tested this idea by assessing whether synaptic plasticity is associated with concurrent changes in axonal conduction within individual neurons, using simultaneous measurements of synaptic strength and axonal conduction latency in hippocampal CA1 pyramidal cells.

## Materials and Methods

### Slice preparation

All animal procedures were conducted in accordance with the National Institutes of Health Guide for the Care and Use of Laboratory Animals and with protocols approved by the Animal Research Committees of Yamagata University. We minimized the number of animals used and their suffering was minimized. Hippocampal slices were obtained from 26- to 32-d-old male Sprague Dawley rats. After the animals were decapitated under deep isoflurane anesthesia, the hippocampus was dissected rapidly from one cerebral hemisphere. To obtain long alveus fibers in a hippocampal slice, 400-µm-thick slices were prepared at a 45° plane to the septotemporal axis using a rotary slicer (DTY-7700; Dosaka). Before recording, the slices were incubated for at least 1 h at 30°C in artificial cerebrospinal fluid (ASCF) containing the following (in mM): 124.0 NaCl, 3.0 KCl, 2.5 CaCl_2_, 2.0 MgCl_2_, 22.0 NaHCO_3_, 1.25 NaH_2_PO_4_, and 10.0 glucose at pH 7.4, aerated with a gas mixture of 95% O_2_ and 5% CO_2_.

### Electrophysiological recordings

For recordings, a slice was transferred to a recording chamber and perfused continuously with ASCF at a rate of 2 ml/min at 30°C; this temperature was selected to maintain stable whole-cell recordings during the prolonged periods required for the simultaneous monitoring of synaptic responses and action potentials while preserving physiological neuronal responsiveness. For whole-cell recordings, pyramidal cells in the hippocampal CA1 region were visualized using an infrared differential interference contrast microscope (E600-FN; Nikon) with a ×40 water immersion objective. Patch electrodes were pulled from borosilicate glass (World Precision Instruments) using a micropipette puller (P-97; Sutter Instrument). The electrodes were filled with an internal solution containing the following (in mM): 140 K-gluconate, 10 HEPES, 0.5 EGTA, 10 NaCl, 1 MgCl_2_, 2 Mg-ATP, and 0.2 Na-GTP, adjusted to pH 7.3 with KOH. For experiments examining the role of intracellular Ca^2+^, EGTA in the internal solution was replaced with 10 mM BAPTA. In an additional set of experiments, the concentration of K-gluconate was reduced accordingly to compensate for the potassium introduced by BAPTA and to maintain a comparable nominal potassium concentration; 40 mM sucrose was then added to adjust osmolality. The osmolality of the standard internal solution was ∼285–300 mOsm/kg. The BAPTA-containing internal solution prepared with BAPTA tetrapotassium had an osmolality of ∼320–330 mOsm/kg, whereas the potassium- and osmolality-adjusted BAPTA-containing internal solution had an osmolality of ∼290 mOsm/kg. The resistance of the electrodes was 5–7 MΩ. Whole-cell recordings were obtained from the visually identified soma of CA1 pyramidal cells in current-clamp mode at near resting membrane potential (−58 to −67 mV). Synaptic and axonal responses were evoked using stimulation electrodes placed in the Schaffer collateral pathway and the alveus between the CA1 region and subiculum, where the axons from CA1 pyramidal cells pass through to reach their target subicular neurons. Orthodromic stimulation was used to elicit excitatory postsynaptic potentials (EPSPs), whereas the alveus was stimulated to evoke antidromic action potentials (Anti-APs) in the same recorded neuron. To obtain stable and reliable EPSP recordings, the GABA_A_ receptor antagonist bicuculline (1 μM) was bath applied to partially suppress inhibitory synaptic transmission. Bicuculline was dissolved in distilled water and diluted in ASCF before use. The concentration of bicuculline was selected based on previous studies demonstrating its concentration-dependent effects in hippocampal pyramidal neurons (Shirasaki et al., 1991) and to minimize excessive network disinhibition while maintaining stable network activity during prolonged recordings. In a subset of experiments, AMPA, NMDA, and GABA_A_ receptors were blocked by bath application of 6,7-dinitroquinoxaline-2,3-dione (DNQX; 20 µM), d,ʟ-(−)-2-amino-5-phosphonopentanoic acid (AP5; 50 µM), and bicuculline (10 µM), respectively, to evaluate the potential contribution of synaptic activation to antidromic latency measurements. Antidromic waveforms and latencies were compared before and after receptor blockade using identical stimulation and recording conditions. EPSPs and Anti-APs were recorded in alternating blocks of 10 responses each, with all stimuli delivered at uniform 15 s intervals throughout the experiment, including between successive blocks, unless otherwise noted. Synaptic efficacy was quantified by measuring the initial EPSP slope, calculated from the linear portion of the rising phase of each response. Axonal conduction properties were assessed by measuring the latency of Anti-APs, defined as the time interval between the stimulus artifact and the start of the action potential. The start of the action potential was taken as the time when the slope of the tangent to the action potential became >10 mV/ms (Yamazaki et al., 2014). To visualize the time course, Anti-AP latencies were plotted as individual responses in 15 s intervals or as block-averaged values of 10 consecutive responses, as indicated in each figure. Block-averaged Anti-AP latencies were used for statistical analysis. Baseline values for the EPSP slope and Anti-AP latency were established from the initial 10 min recording period prior to any experimental manipulation and were defined as 100%, unless otherwise noted. Except for representative examples shown in absolute units, all subsequent measurements were expressed as a percentage of this baseline. For some analyses, values were additionally expressed as percentage change from baseline to facilitate direct comparisons across conditions. Recording stability was monitored throughout the experiments by applying small hyperpolarizing current steps and examining the resulting voltage responses, together with the continuous monitoring of action potential amplitudes and waveforms. Recordings were discontinued or excluded from analysis if these measures changed by >25%, indicating a deterioration of recording quality. Series resistance was not compensated for during recordings. LTP was induced using a high-frequency stimulation (HFS) protocol consisting of 100 pulses delivered at 100 Hz to Schaffer collaterals, paired with a 1 s depolarizing current injection into the recorded neuron sufficient to elicit 20–30 action potentials. Although resting membrane potential values in some recordings were relatively depolarized, the recordings included in the analysis remained stable throughout the experiments, Anti-APs were elicited reliably, and analyses were based primarily on within-cell comparisons relative to baseline values. Current responses were recorded using Axopatch-200B or Axopatch-1D amplifiers (Axon Instruments), filtered (5 kHz), and stored in a computer after conversion (digitized at 50–100 kHz) by an analog-digital converter (PCI-6023E; National Instruments). Data were analyzed off-line using a wave analysis program developed by ourselves and OriginPro (OriginLab). Drugs were obtained from the following sources: bicuculline methobromide (methobromide salt of [+]-bicuculline) and BAPTA (Wako Pure Chemical); DNQX and AP5 (Sigma-Aldrich).

### Experimental design and statistical analysis

All data are expressed as the mean ± standard error of the mean. Sample size (*n*) refers to the number of neurons analyzed in electrophysiological recordings. Sample sizes were not determined by formal a priori power calculations. Experiments with limited sample sizes that were performed as confirmatory control experiments were summarized descriptively rather than analyzed using inferential statistical tests. Accordingly, these datasets are presented as individual data points together with descriptive statistics. Assumptions of normality were assessed using the Shapiro–Wilk test for datasets analyzed with parametric statistical methods. To analyze HFS-induced synaptic and axonal changes, the magnitude of synaptic potentiation was quantified as the mean EPSP slope measured at 50–52 min after HFS to assess stabilized LTP, whereas axonal conduction changes were quantified as the mean Anti-AP latency measured during a representative post-HFS time window (23–25 min), corresponding to the period in which latency shortening consistently reached maximal or near-maximal levels in group-averaged time courses. These values were compared with corresponding measurements obtained from no-HFS control recordings using an unpaired two-tailed Student's *t* test. To assess the stability of recordings under control conditions without HFS, EPSP slope and Anti-AP latency values measured during the late recording period (EPSP: 58–60 min; Anti-AP: 55–57 min) were compared with baseline values (defined from the initial 10 min) using paired two-tailed Student's *t* tests. To examine the relationship between synaptic and axonal plasticity, Pearson's correlation analysis was used to assess the association between changes in synaptic efficacy and changes in Anti-AP latency. Synaptic efficacy was quantified as the mean EPSP slope measured during defined post-HFS time windows (early: 0–2 min; intermediate: 25–27 min; late: 45–47 min). For analyses requiring a single quantitative measure of axonal conduction plasticity, the magnitude of latency change was defined as the maximum percentage change in Anti-AP latency calculated from block-averaged values (each block comprising 10 responses) during the post-HFS recording period. This value was used for correlation analyses and for comparisons across experimental conditions. For experiments involving the blockade of LTP induction, intracellular Ca^2+^ buffering (BAPTA) or pharmacological inhibition of NMDA receptors (AP5) was applied. Under these conditions, changes in synaptic efficacy were quantified as the mean EPSP slope measured at 45–47 min after HFS, and axonal conduction plasticity was quantified using the same latency-based metric defined above. Data obtained under the BAPTA or AP5 condition were compared with those obtained under the standard HFS condition (EGTA-containing internal solution without pharmacological blockade). Since group sizes differed and equality of variance could not be assumed, Welch's two-tailed *t* test was used for these comparisons. To examine the spatial dependence of axonal conduction plasticity, baseline Anti-AP latency measured prior to HFS was used as a proxy for the relative distance between the soma and axonal stimulation site. For each recording, two temporal parameters were quantified: latency onset time, defined as the first time point at which a consistent reduction in latency exceeding 2% from baseline was observed across successive measurements, and latency-to-peak time, defined as the time required to reach the maximum latency reduction following HFS. Pearson's correlation analysis was used to assess the relationships between baseline latency and latency onset time and latency-to-peak time. A difference was considered statistically significant at *p* < 0.05.

## Results

### LTP induction is associated with a delayed enhancement of axonal conduction

**Figure 1.**
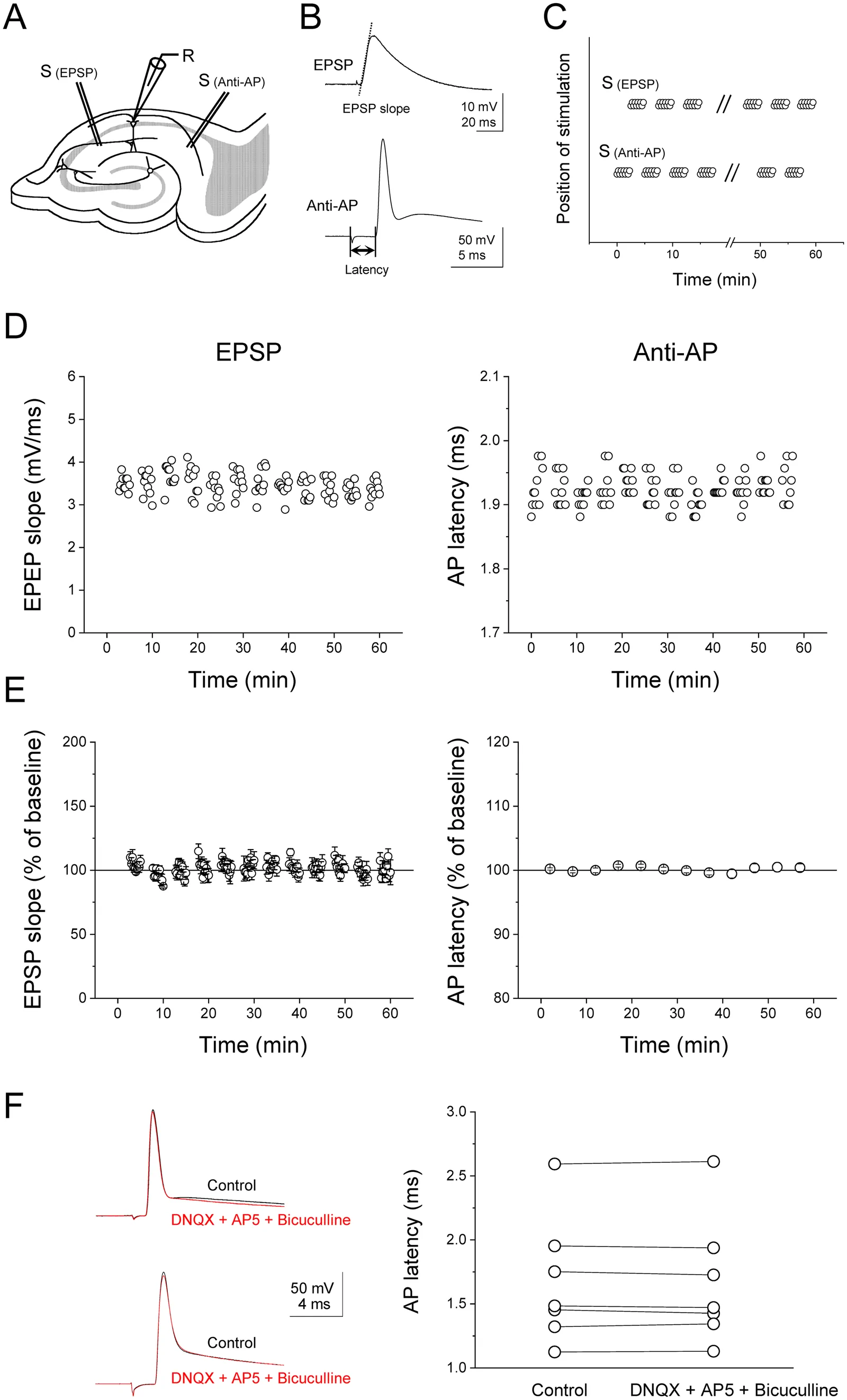
Synaptic and axonal responses remain stable during alternating stimulation. ***A***, Experimental configuration for the simultaneous assessment of synaptic and axonal function in hippocampal CA1 pyramidal cells. Whole-cell recordings were obtained from CA1 pyramidal cells. Excitatory postsynaptic potentials (EPSPs) were evoked by stimulation of Schaffer collateral afferents, and antidromic action potentials (Anti-APs) were elicited by axonal stimulation of the recorded neuron. ***B***, Representative traces illustrating the quantification of synaptic and axonal responses. Synaptic efficacy was assessed by measuring the initial EPSP slope, and axonal conduction was assessed by measuring Anti-AP latency. ***C***, Recording protocol used to assess baseline stability. EPSPs and Anti-APs were recorded in alternating blocks of 10 responses each, with all stimuli delivered at uniform 15 s intervals. This alternating protocol was maintained for 60 min in the absence of conditioning stimulation. ***D***, Representative time course of the EPSP slope (left, mV/ms) and Anti-AP latency (right, ms) during a control recording without conditioning stimulation. ***E***, Summary plots of the EPSP slope (left) and Anti-AP latency (right) during control recordings without conditioning stimulation. Data from individual neurons (*n* = 6) were normalized to the baseline period (first 10 min) and plotted as mean ± standard error of the mean. There were no significant changes in either synaptic or axonal measures over the 60 min recording period. ***F***, Validation of antidromic latency measurements under synaptic receptor blockade. Left, Representative antidromic responses recorded before and after the application of DNQX, AP5, and bicuculline from proximal and distal stimulation sites, shown as examples with relatively short and long antidromic latencies, respectively. Right, Paired comparison of antidromic latency before and after receptor blockade in individual neurons, including recordings obtained with proximal and distal stimulation (*n* = 7).

To investigate how action potential latency changes in neurons undergoing LTP, we performed whole-cell recordings from pyramidal cells in the hippocampal CA1 region. EPSPs were evoked by stimulating Schaffer collateral afferent fibers, and Anti-APs were elicited by stimulating the axons of the recorded neurons (Fig. 1*A*). Synaptic and axonal responses were quantified by measuring the EPSP slope and Anti-AP latency, respectively (Fig. 1*B*). To evaluate the stability of these responses and to confirm that alternating the two types of stimulation did not introduce cross-interference, recordings were initially conducted without applying any conditioning stimuli such as HFS. In these control sessions, recordings were performed in alternating blocks of 10 EPSPs and 10 Anti-APs, with stimuli delivered at 15 s intervals, and this protocol was maintained for 60 min (Fig. 1*C*). The first 10 min of recordings were used to establish the baseline (defined as 100%) for each response. EPSP slope values measured at 58–60 min averaged 99.3 ± 5.3% of baseline (*n* = 6) and did not differ significantly from baseline (*t*_(5)_ = 0.50, *p* = 0.69, paired *t* test; Fig. 1*D*,*E*). Similarly, Anti-AP latency measured at 55–57 min was 100.4 ± 0.78% of baseline (*n* = 6), with no significant difference from baseline (*t*_(5)_ = 0.70, *p* = 0.51, paired *t* test; Fig. 1*D*,*E*). These results indicate that both responses remained stable throughout the recording session and that alternating stimulation did not introduce detectable drift or cross-interference between synaptic and axonal responses. Since alveus stimulation can evoke synaptically mediated components in addition to Anti-APs, we examined whether synaptic transmission contributed to the antidromic latency measurements. In recordings with relatively short antidromic latencies, a slow depolarizing component following an Anti-AP was partially reduced by blockade of AMPA, NMDA, and GABA_A_ receptors with DNQX, AP5, and bicuculline, respectively, indicating that synaptically mediated responses could contribute to the later portions of the recorded waveforms. However, the sharp onset of the Anti-AP and the measured latency were essentially unchanged by receptor blockade. In recordings with longer antidromic latencies, receptor blockade had little effect on the waveforms and did not alter latency. These findings indicate that, although synaptic activation can contribute to later waveform components under some conditions, the latency measurements used in the present study primarily reflect antidromic spike propagation and are minimally influenced by synaptic activation (Fig. 1*F*).

**Figure 2.**
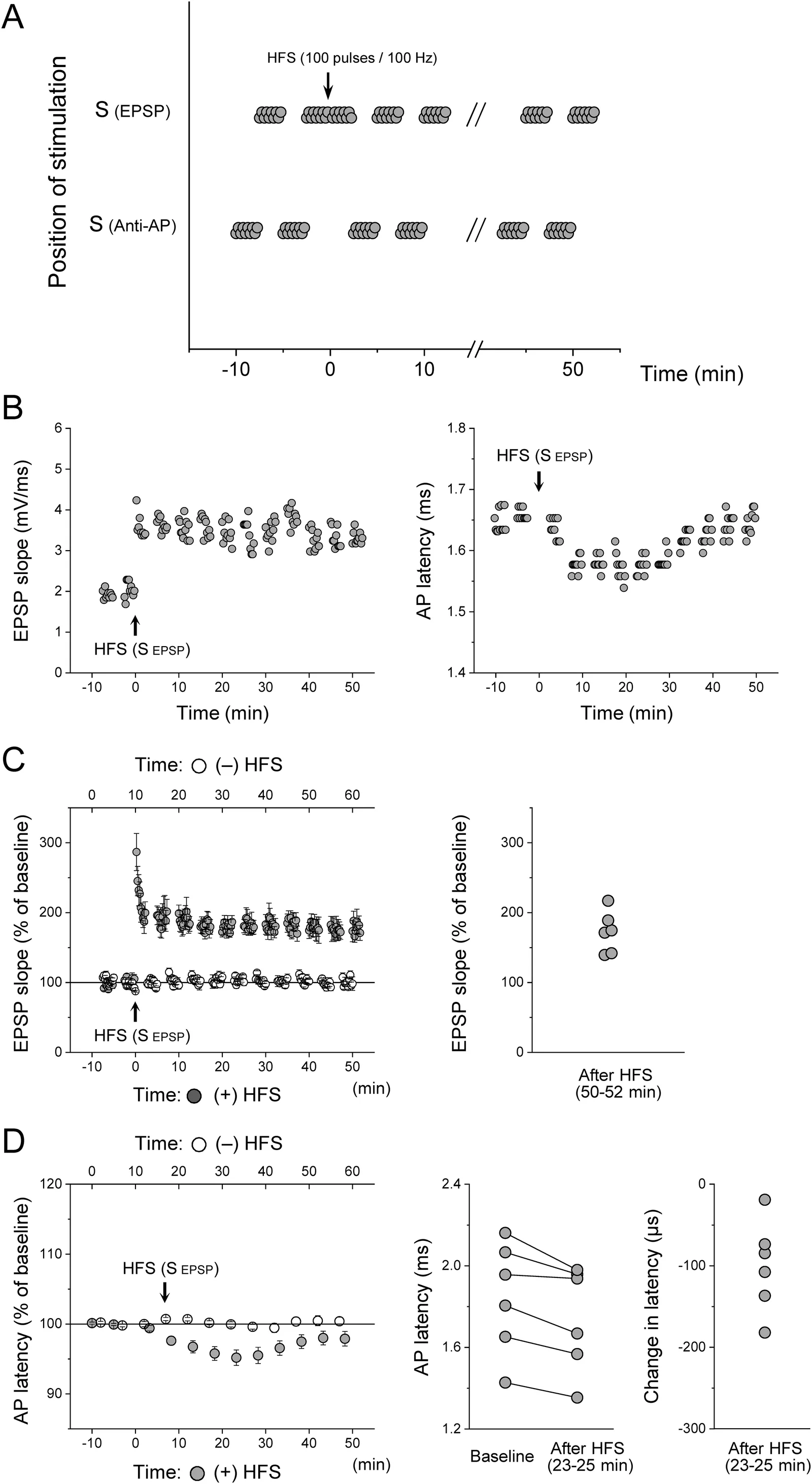
Antidromic action potential (Anti-AP) latency decreases with a delayed time course following long-term potentiation (LTP) induction. ***A***, Experimental protocol. High-frequency stimulation (HFS; 100 pulses at 100 Hz) was delivered to Schaffer collaterals in conjunction with a 1 s depolarizing current injection into the recorded CA1 pyramidal cell. Ten excitatory postsynaptic potentials (EPSPs) were recorded immediately before and after HFS, after which recordings resumed in alternating blocks of 10 EPSPs and 10 Anti-APs at 15 s intervals. ***B***, Representative example showing the time course of the EPSP slope (left) and Anti-AP latency (right) from the same neuron. ***C***, Left, Summary time course of the normalized EPSP slope averaged across neurons. Data from no-HFS control recordings (Fig. 1*E*) are shown for comparison. Right, Individual EPSP slope values measured at 50–52 min after HFS. Each symbol represents one neuron (*n* = 6). ***D***, Left, Summary time course of normalized Anti-AP latency averaged across neurons. Data from no-HFS control recordings (Fig. 1*E*) are shown for comparison. Middle, Paired plots of actual Anti-AP latency values measured before HFS (Baseline) and after HFS (23–25 min) for individual neurons. Lines connect paired measurements obtained from the same neuron. Right, Corresponding changes in latency values (After HFS − Baseline) for each neuron. Negative values indicate latency shortening following HFS. Data are presented as mean ± standard error of the mean.

Next, HFS consisting of 100 pulses at 100 Hz was applied to the Schaffer collaterals in conjunction with a 1 s depolarizing current injection into the recorded neuron to assess whether LTP could be induced under a similar stimulation protocol. Specifically, 10 consecutive EPSPs were recorded immediately before and after HFS, after which the alternating pattern of 10 EPSPs and 10 Anti-APs was resumed (Fig. 2*A*). Even with this intermittent recording condition, a robust increase in EPSP slope was observed (50–52 min after HFS; 172.1 ± 12.4% of baseline, *n* = 6, *t*_(10)_ = 6.12, *p* < 0.001 vs no-HFS control; Fig. 2*B*,*C*), indicating the successful induction of LTP. We then examined how Anti-AP latency evolved following HFS in the same neuron. Notably, there was no immediate change in antidromic latency following HFS; however, a progressive reduction in latency began to emerge at several minutes after stimulation, reaching its peak at 23–25 min post-HFS (95.2 ± 1.1% of baseline, *n* = 6, *t*_(10)_ = 4.09, *p* = 0.0022 vs no-HFS control; Fig. 2*B*,*D*). To illustrate the magnitude and consistency of the observed latency changes across individual neurons, paired plots of the actual Anti-AP latency values measured before and after HFS (23–25 min) were analyzed (Fig. 2*D*). Although the absolute latency shifts were small, latency shortening was observed consistently across recordings, and the corresponding changes in latency values demonstrated a systematic reduction in antidromic latency following HFS (−100.6 ± 22.8 µs, *n* = 6; 95% CI, −159 to −42 µs; Fig. 2*D*). This reduction was transient, with latency values gradually returning toward baseline over the subsequent recording period.

### Latency reduction scales with the magnitude of synaptic potentiation

**Figure 3.**
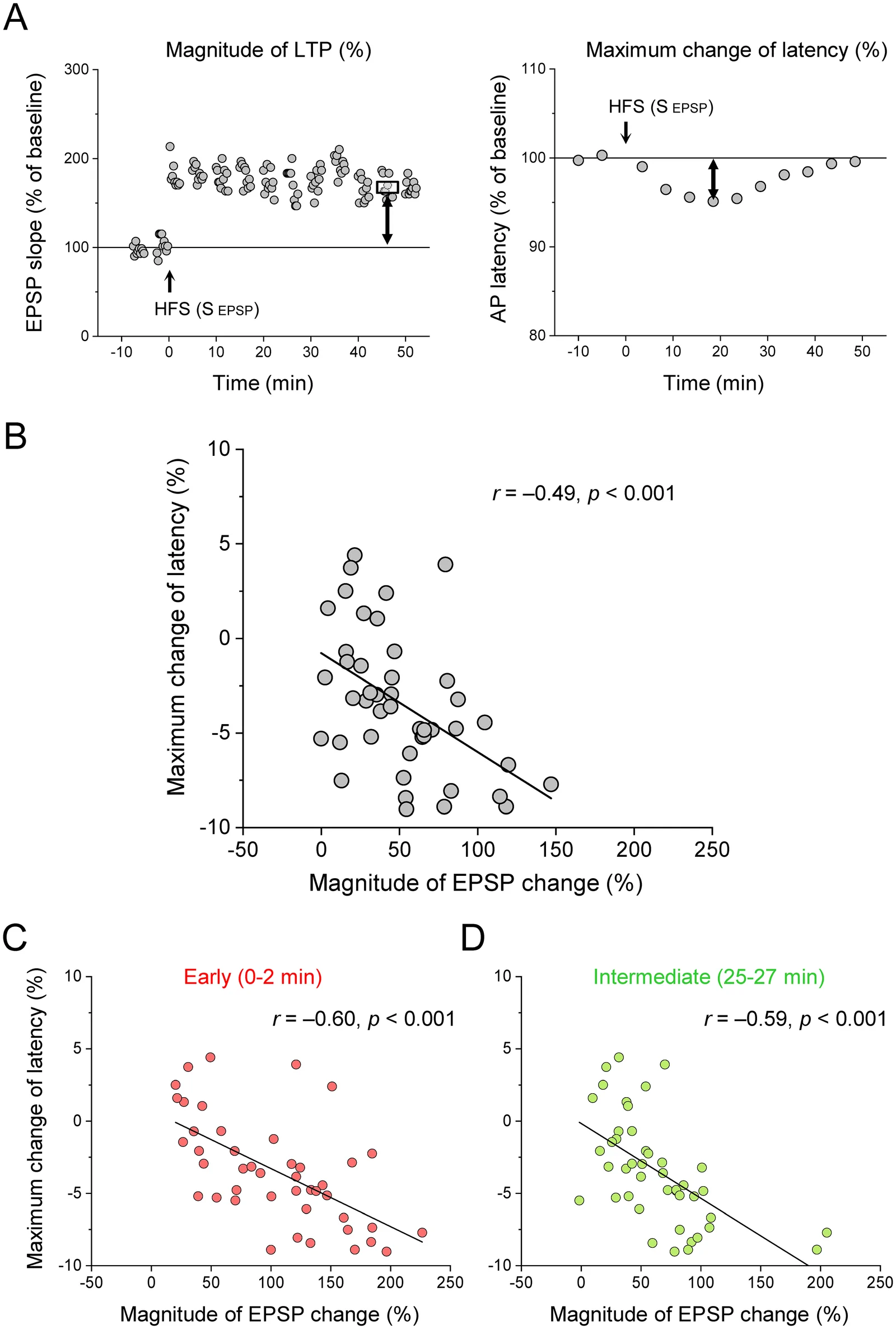
Axonal conduction changes correlate with the magnitude of synaptic potentiation following high-frequency stimulation (HFS). ***A***, Time courses of the excitatory postsynaptic potential (EPSP) slope (left) and antidromic action potential (Anti-AP) latency (right) from a representative neuron, illustrating the parameters used for correlation analysis. The EPSP slope time course (left) is identical to that shown in Figure 2*B* and highlights the late post-HFS period (45–47 min) used to quantify synaptic potentiation. The Anti-AP latency trace (right) is derived from the same recording shown in Figure 2*B* but replotted as averages of consecutive blocks of 10 responses to illustrate the extraction of the maximal latency change used for analysis. ***B***, Scatter plot showing the relationship between the magnitude of late phase synaptic potentiation (EPSP slope at 45–47 min post-HFS) and the maximum change in Anti-AP latency (*n* = 44). A significant negative correlation was observed, indicating that stronger synaptic potentiation was associated with greater reductions in antidromic latency. ***C***, ***D***, Correlation analyses between changes in the EPSP slope and Anti-AP latency using EPSP slopes measured during the early (0–2 min; ***C***) and intermediate (25–27 min; ***D***) post-HFS periods. There were significant negative correlations at both time points. Each data point represents a single neuron. Pearson's correlation coefficients and corresponding *p* values are indicated in each panel.

The delayed and time-dependent reduction in antidromic latency following HFS raised the question of whether this axonal change is functionally linked to the expression of synaptic plasticity in the same neuron. To address this, we examined the relationship between the magnitude of LTP and the extent of latency reduction in Anti-APs, under the hypothesis that axonal conduction plasticity may reflect the degree of synaptic strengthening. The magnitude of LTP was quantified as the mean EPSP slope recorded at 45–47 min after HFS (Fig. 3*A*). For latency analysis, antidromic responses were averaged across each set of 10 stimuli, as described above, and the maximal reduction in latency was extracted for each experiment (Fig. 3*A*). Using percentage change values, we assessed the correlation between LTP magnitude and the degree of latency reduction. A significant negative correlation was observed (*n* = 44, *r* = −0.49, *p* < 0.001), indicating that stronger synaptic potentiation tended to be associated with a greater enhancement of axonal conduction (Fig. 3*B*). Although latency reduction predominated across the population, a small number of recordings showed little change or slight increases in latency, indicating variability in the expression of latency modulation across individual cells. To determine whether this relationship was specific to the late phase of synaptic potentiation or was also present at earlier stages following HFS, we extended this analysis to include EPSP slopes measured at two additional time windows: an early phase (0–2 min) and an intermediate phase (25–27 min) after HFS. Significant negative correlations were observed at both time points (early: *r* = −0.60, *p* < 0.001; intermediate: *r* = −0.59, *p* < 0.001; Fig. 3*C*,*D*). Although the correlation coefficient was largest at the early time point, strong correlations were also observed during the intermediate phase, a period that temporally overlapped with the emergence and peak of antidromic latency reduction. The changes in antidromic latency continued to evolve over tens of minutes following HFS, whereas early EPSP enhancement occurred immediately after stimulation. Importantly, a robust and statistically significant correlation persisted when LTP magnitude was assessed during the late phase, when synaptic potentiation had stabilized. Thus, while the correlations observed at the early time points may partly reflect transient, activity-dependent synaptic changes, the persistence of a significant correlation at the later time points supports the idea that axonal conduction plasticity is not limited to acute poststimulation activity but is also associated with enduring synaptic potentiation.

### Postsynaptic Ca^2+^ signaling is required for axonal conduction plasticity

**Figure 4.**
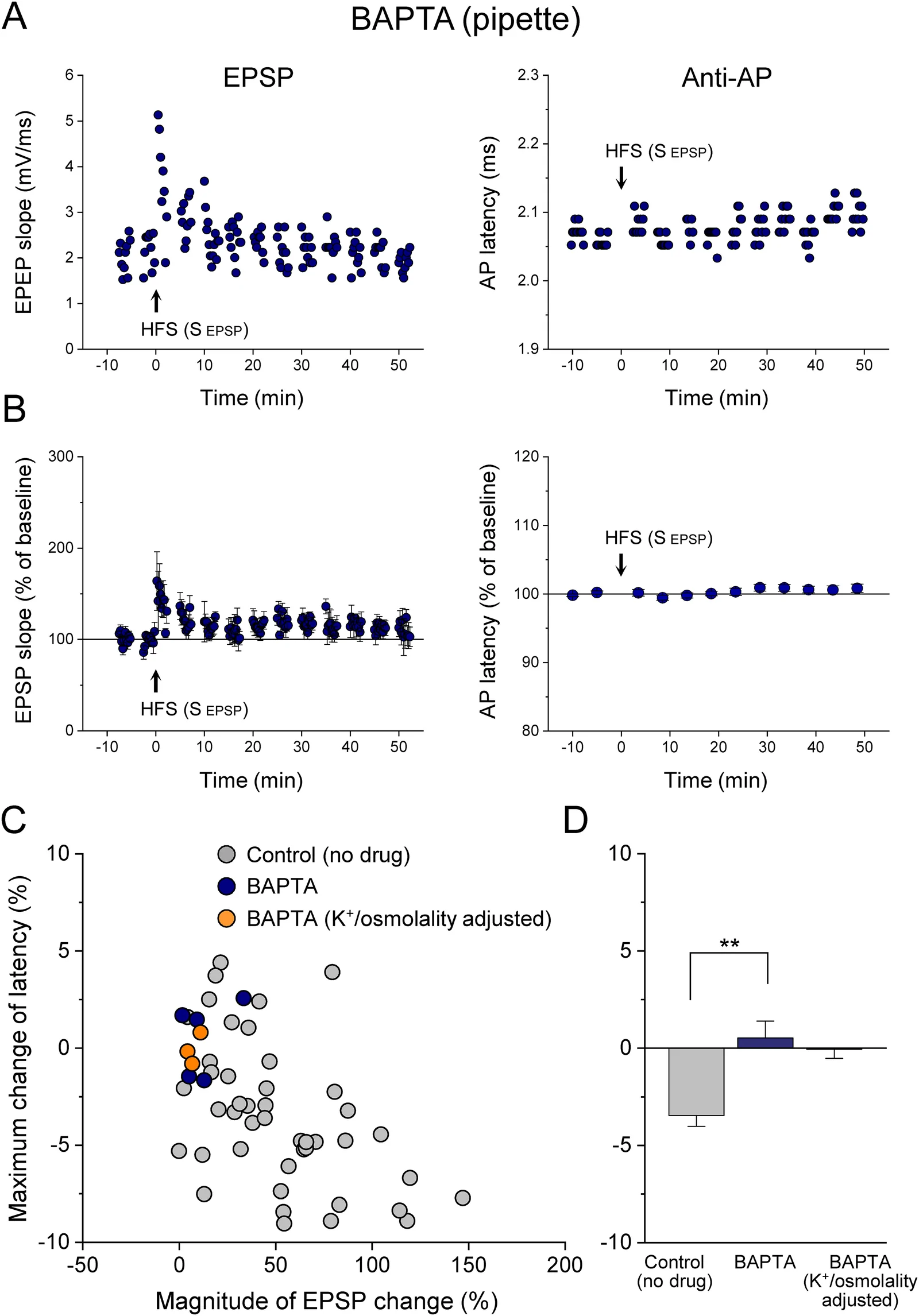
Intracellular Ca^2+^ buffering with BAPTA abolishes synaptic potentiation and antidromic latency changes. ***A***, Representative recording from a neuron filled with 10 mM BAPTA showing the time courses of the excitatory postsynaptic potential (EPSP) slope (left) and antidromic action potential (Anti-AP) latency (right). High-frequency stimulation (HFS; arrow) failed to induce a sustained increase in the EPSP slope and did not produce a detectable reduction in Anti-AP latency. ***B***, Group data showing normalized time courses of the EPSP slope (left) and Anti-AP latency (right) following HFS in BAPTA-filled neurons (*n* = 5). Under intracellular Ca^2+^ buffering, neither synaptic potentiation nor antidromic latency shortening was observed. ***C***, Scatter plot showing the relationship between the change in synaptic efficacy (mean EPSP slope measured at 45–47 min after HFS) and maximum Anti-AP latency change under intracellular Ca^2+^ buffering with BAPTA. Data obtained with the BAPTA-containing internal solution (*n* = 5) and with the potassium- and osmolality-adjusted BAPTA-containing internal solution (*n* = 3) are shown. Each point represents an individual neuron. For direct comparison, data from the standard HFS condition shown in Figure 3*B* are replotted in the background. ***D***, Summary of the maximum change in Anti-AP latency following HFS under the BAPTA condition (*n* = 5) and adjusted BAPTA condition (*n* = 3), compared with the standard HFS condition. Bars represent mean ± standard error of the mean. **p < 0.01 (Welch's *t* test).

Having established a significant relationship between the magnitude of synaptic potentiation and the extent of antidromic latency reduction, we next asked whether this association reflects a causal dependence on LTP induction mechanisms. To address this question, we examined the effects of intracellular Ca^2+^ buffering on synaptic potentiation and axonal conduction plasticity. In a separate series of experiments, the patch pipette solution was modified to contain 10 mM BAPTA, a fast Ca^2+^ chelator, in place of the 0.5 mM EGTA used in the standard recording condition. This manipulation effectively prevented the rise in postsynaptic intracellular Ca^2+^ required for LTP induction (Malenka et al., 1988; Bliss and Collingridge, 2013). Under these conditions, HFS failed to induce synaptic potentiation, with the EPSP slope at 45–47 min post-HFS remaining at 112.2 ± 5.7% of baseline (*n* = 5, *t*_(12.93)_ = 5.06, *p* < 0.001 vs standard HFS condition, Welch's *t* test; Fig. 4*A*,*B*). Notably, the changes in axonal conduction were also abolished: the peak latency shift was only +0.52 ± 0.86%, indicating no significant reduction in antidromic latency (*n* = 5, *t*_(7.86)_ = 3.86, *p* = 0.0050 vs standard HFS condition, Welch's *t* test; Fig. 4*A*–*D*). To control for the increase in intracellular potassium associated with the tetrapotassium form of BAPTA, additional experiments were performed using a potassium- and osmolality-adjusted BAPTA-containing internal solution in which the concentration of K-gluconate was reduced accordingly and osmolality was adjusted with sucrose. Under these conditions, HFS likewise failed to induce stable synaptic potentiation, with the EPSP slope at 45–47 min post-HFS remaining at 107.3 ± 2.0% of baseline (*n* = 3), and there was no significant reduction in antidromic latency (peak latency shift: −0.06 ± 0.5%, *n* = 3; Fig. 4*C*,*D*). These results were comparable to those obtained with the original BAPTA-containing internal solution, indicating that the observed effects were not attributable to differences in intracellular potassium concentration or osmolality. Together, these findings support the view that the observed reduction in antidromic latency is not a nonspecific effect of stimulation, but rather a plastic change that depends on the successful induction of LTP in postsynaptic neuron.

**Figure 5.**
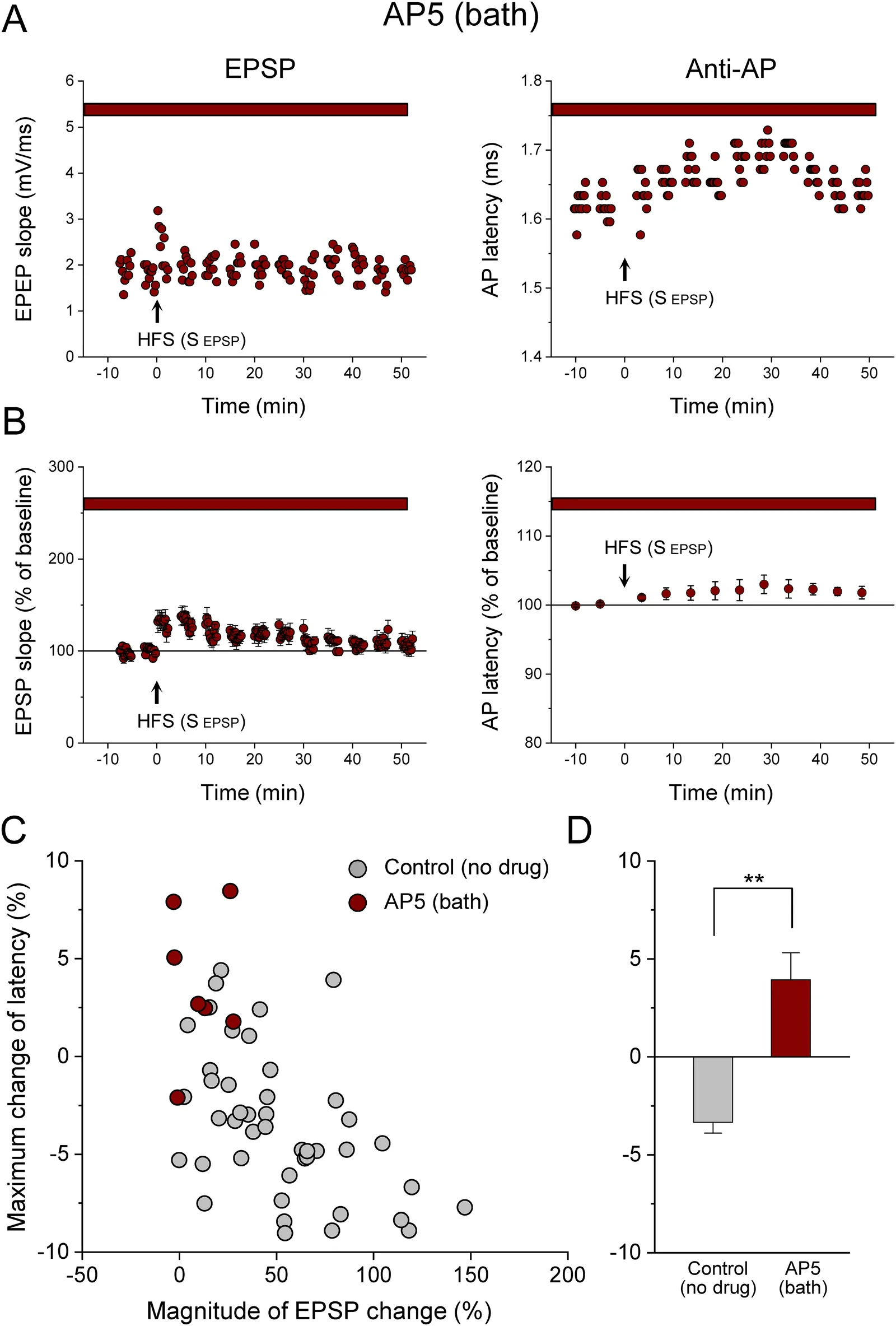
NMDA receptor blockade prevents synaptic potentiation and reveals latency prolongation following high-frequency stimulation (HFS). ***A***, Representative example showing the time course of the excitatory postsynaptic potential (EPSP) slope (left) and antidromic action potential (Anti-AP) latency (right) recorded from a neuron in the presence of the NMDA receptor antagonist AP5. HFS (arrow) failed to induce sustained synaptic potentiation, and Anti-AP latency was not shortened but instead showed a small prolongation. ***B***, Group data showing the time course of the normalized EPSP slope (left) and Anti-AP latency (right) under AP5 treatment (*n* = 7). The EPSP slope remained near baseline throughout the post-HFS period, indicating the blockade of NMDA receptor-dependent synaptic potentiation. In contrast, Anti-AP latency showed no clear shortening over time. ***C***, Scatter plot showing the relationship between changes in synaptic efficacy (mean EPSP slope measured at 45–47 min after HFS) and the maximum change in Anti-AP latency under the AP5 condition, overlaid on data from the standard HFS condition (same dataset as in Fig. 3*B*) for comparison. ***D***, Summary of the maximum change in Anti-AP latency following HFS under the AP5 condition (*n* = 7), compared with the standard HFS condition. Anti-AP latency exhibited a modest but significant increase under AP5. Bars represent mean ± standard error of the mean. **p < 0.01 (Welch's *t* test).

We next examined whether pharmacological blockade of NMDA receptors would similarly affect the relationship between LTP and changes in axonal conduction. To this end, the NMDA receptor antagonist AP5 (50 µM) was bath applied throughout the experiment to prevent the induction of LTP. As expected, HFS in the presence of AP5 failed to elicit significant synaptic potentiation, with the EPSP slope at 45–47 min post-HFS remaining at 108.6 ± 4.5% of baseline (*n* = 7, *t*_(23.04)_ = 5.71, *p* < 0.001 vs standard HFS condition; Welch's *t* test), consistent with the blockade of NMDA receptor-dependent LTP (Fig. 5*A*,*B*). In contrast to standard HFS conditions, Anti-APs recorded under AP5 treatment did not exhibit latency shortening; instead, a small but significant prolongation of latency was observed (+3.9 ± 1.9%, *n* = 7, *t*_(8.08)_ = 4.96, *p* = 0.0011 vs standard HFS condition; Welch's *t* test; Fig. 5*A*–*D*). To determine whether NMDA receptor blockade alone affected axonal conduction, we examined antidromic latency in the presence of AP5 without HFS. Bath application of AP5 in the absence of HFS produced no detectable change in latency (99.3 ± 0.46%, *n* = 7, *t*_(6)_ = 1.44, *p* = 0.20, paired *t* test; data not shown). Together, these results indicate that NMDA receptor-dependent signaling is required not only for synaptic potentiation but also for the associated facilitation of axonal conduction. Moreover, when NMDA receptor-mediated processes are blocked during strong synaptic activation, axonal conduction can shift toward a transient slowing rather than facilitation.

**Figure 6.**
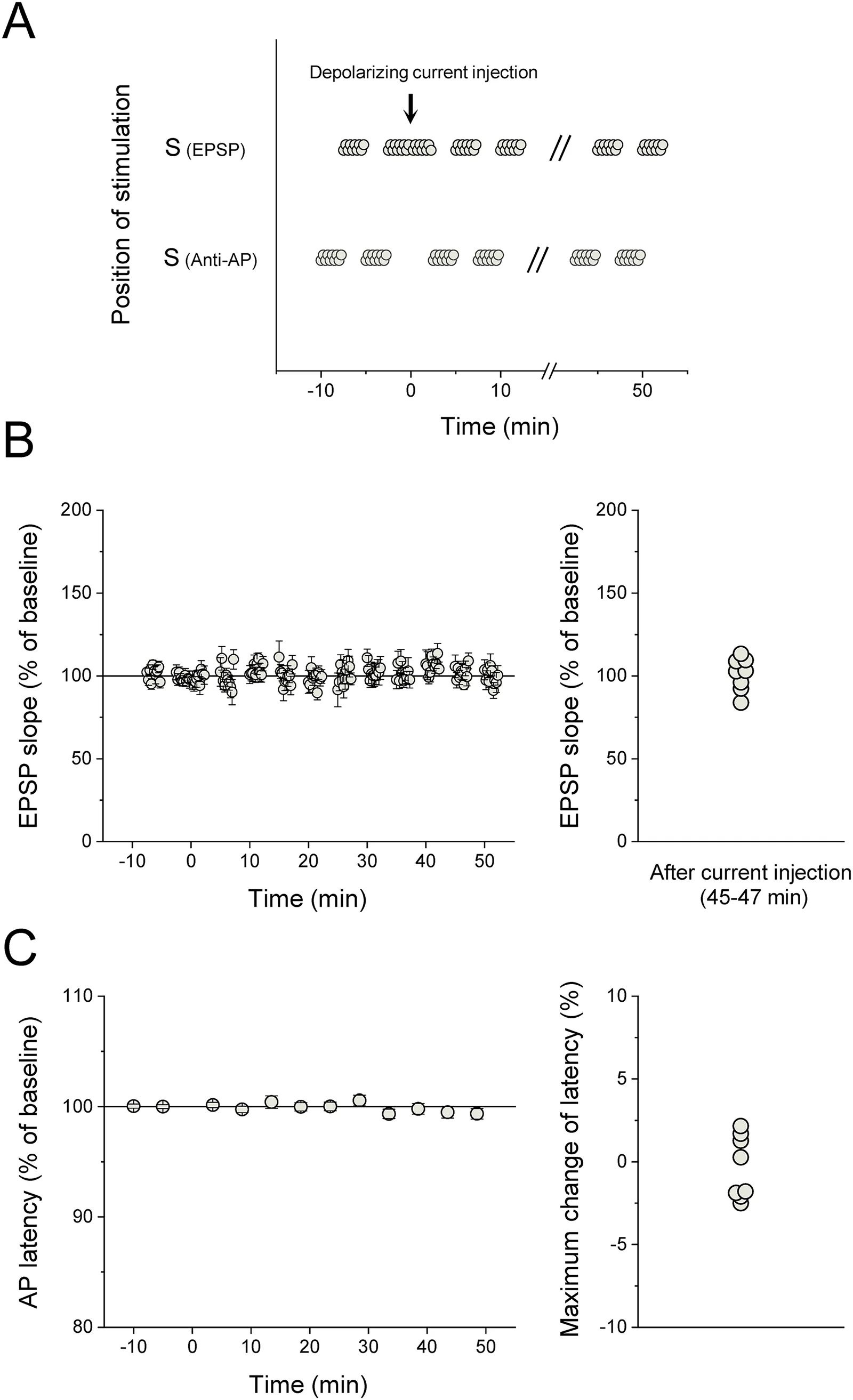
Repetitive postsynaptic firing alone is insufficient to induce changes in antidromic latency. ***A***, Schematic illustration of the control stimulation protocol. A 1 s depolarizing current injection was applied to the recorded neuron to evoke repetitive action potential firing, reproducing the duration and firing intensity of the current injection used during high-frequency stimulation (HFS), but without afferent stimulation. ***B***, Left, Summary time course of changes in synaptic efficacy, measured as excitatory postsynaptic potential (EPSP) slope, following postsynaptic current injection alone. Right, Individual EPSP slope values measured at 45–47 min after current injection. ***C***, Left, Summary time course of antidromic action potential (Anti-AP) latency following postsynaptic current injection alone. Right, Individual maximum changes in Anti-AP latency. Each symbol represents one neuron (*n* = 8). Neither the EPSP slope nor Anti-AP latency exhibited significant changes relative to baseline. Data are presented as mean ± standard error of the mean.

Taken together, these results demonstrate that HFS induces a delayed but pronounced reduction in antidromic latency under conditions that also support the induction of LTP in the same neurons. However, because the HFS protocol necessarily involves strong postsynaptic depolarization and repetitive firing, it remains possible that such activity itself could contribute to the observed changes in latency, independently of synaptic input. To test this possibility, we performed a control experiment in which depolarizing current injection was applied to evoke repetitive spiking for 1 s in the recorded neuron without afferent stimulation (Fig. 6*A*). This protocol reproduced the duration and firing intensity of that used during HFS but did not engage synaptic input. Under these conditions, neither the EPSP slope (101.9 ± 9.9% of baseline, *n* = 8; Fig. 6*B*) nor Anti-AP latency (99.6 ± 2.3% of baseline, *n* = 8) showed any detectable change (Fig. 6*C*). These results indicate that repetitive action potential firing alone is insufficient to induce latency changes, further supporting the requirement for synaptic input in triggering axonal conduction plasticity.

### Temporal emergence of axonal conduction modulation depends on axonal distance

**Figure 7.**
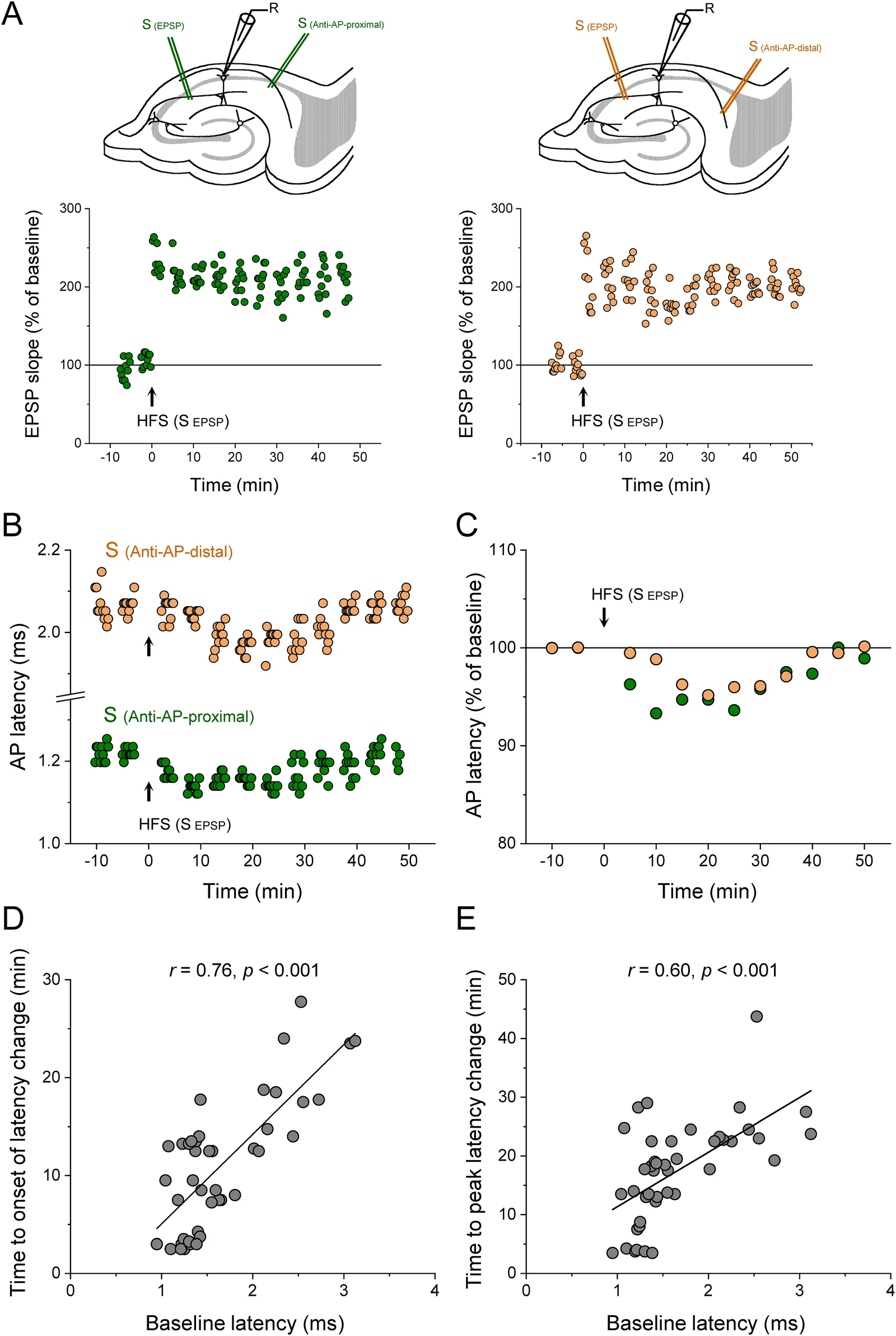
Axonal conduction plasticity exhibits distance-dependent temporal dynamics. ***A***, Representative examples from two neurons to which antidromic stimulation was applied at axonal sites with different baseline latencies. Top, Schematic illustrations showing the relative positions of the recording electrode, the axonal stimulation electrode used to evoke antidromic action potentials (Anti-APs), and the synaptic stimulation electrode used to evoke excitatory postsynaptic potentials (EPSPs). The left and right panels illustrate the configurations yielding short and long baseline antidromic latencies, respectively. Bottom, Corresponding time courses of the EPSP slope recorded from each neuron, demonstrating comparable synaptic potentiation following HFS. ***B***, Time courses of Anti-AP latency recorded from the same neurons shown in ***A***. Despite similar synaptic potentiation, latency shortening emerged earlier at the short-latency (proximal) stimulation site than at the long-latency (distal) site. ***C***, Representative example showing normalized time courses of Anti-AP latency for short- and long-latency stimulation sites. Latency values were averaged within each block of 10 consecutive responses and normalized to the pre-HFS baseline. The short- and long-latency responses are plotted together to illustrate differences in the temporal evolution of latency changes following HFS. ***D***, ***E***, Relationship between baseline antidromic latency and the temporal profile of latency shortening following HFS (*n* = 42). Baseline latency was positively correlated with the onset time of latency reduction (***D***) and the time to peak latency shortening (***E***), indicating that axonal conduction plasticity emerged and matured later at stimulation sites located farther from the soma. Pearson's correlation coefficients and corresponding *p* values are indicated in each panel.

Previous work has demonstrated that the activity-dependent modulation of axonal conduction velocity, associated with oligodendrocyte NKCC1 function, varies along the length of individual axons (Yamazaki et al., 2021). Specifically, the enhancement of conduction velocity induced by oligodendrocyte depolarization differs in magnitude across axonal regions, indicating that axonal conduction plasticity is spatially organized. In light of these findings, and given the association between LTP induction and changes in antidromic latency described above, we examined whether axonal conduction plasticity depends on the site of axonal stimulation. Since the exact trajectory and three-dimensional length of an axon cannot be determined during electrophysiological experiments, the baseline latency of Anti-APs, measured prior to HFS, was used as a proxy for the distance between the soma and axonal stimulation site. By comparing neurons with different baseline latencies, we asked whether the magnitude or temporal profile of latency changes varied with estimated axonal distance under the same LTP induction protocol. To address this question, we first examined representative recordings obtained from neurons stimulated at axonal sites with different baseline latencies, ∼1.2 ms for a relatively proximal site and 2.05 ms for a more distal site (Fig. 7*A*). In both neurons, HFS induced a comparable degree of LTP, as reflected by similar increases in the EPSP slope (Fig. 7*A*). Despite this similarity in synaptic potentiation, the temporal dynamics of the latency changes differed markedly between both conditions. In neuron with a shorter baseline latency (proximal stimulation), a reduction in antidromic latency emerged earlier than in neuron with a longer baseline latency (distal stimulation; Fig. 7*B*). These differences were clearly visualized by plotting the time course of latency changes, with each data point representing the average of 10 consecutive antidromic responses normalized to baseline (Fig. 7*C*). To assess the spatial dependence of axonal conduction plasticity systematically, we extended this analysis across neurons with a broad range of baseline latencies. For each recording, two temporal parameters were quantified: latency onset time, defined as the time point at which a consistent decrease in antidromic latency exceeding 2% from baseline was first observed across successive measurements, and latency-to-peak time, defined as the time required to reach the maximum reduction in latency. When these parameters were plotted against baseline latency, a clear and systematic relationship emerged (*n* = 42), i.e., the onset and peak of latency shortening occurred earlier in neurons with shorter baseline latencies, corresponding to stimulation sites located closer to the soma (Fig. 7*D*,*E*). Quantitative analysis confirmed this observation, revealing a strong positive correlation between baseline latency and latency onset time (*r* = 0.76, *p* < 0.001; Fig. 7*D*), as well as a significant correlation between baseline latency and latency-to-peak time (*r* = 0.60, *p* < 0.001; Fig. 7*E*). Together, these results indicate that the farther the stimulation site is from the soma, as estimated by a longer baseline latency, the more delayed the emergence and maturation of HFS-induced axonal conduction changes. This spatial dependence suggests that the temporal profile of axonal conduction plasticity is shaped by the functional, i.e., electrotonic or physiological distance between the soma and site of axonal activation.

**Figure 8.**
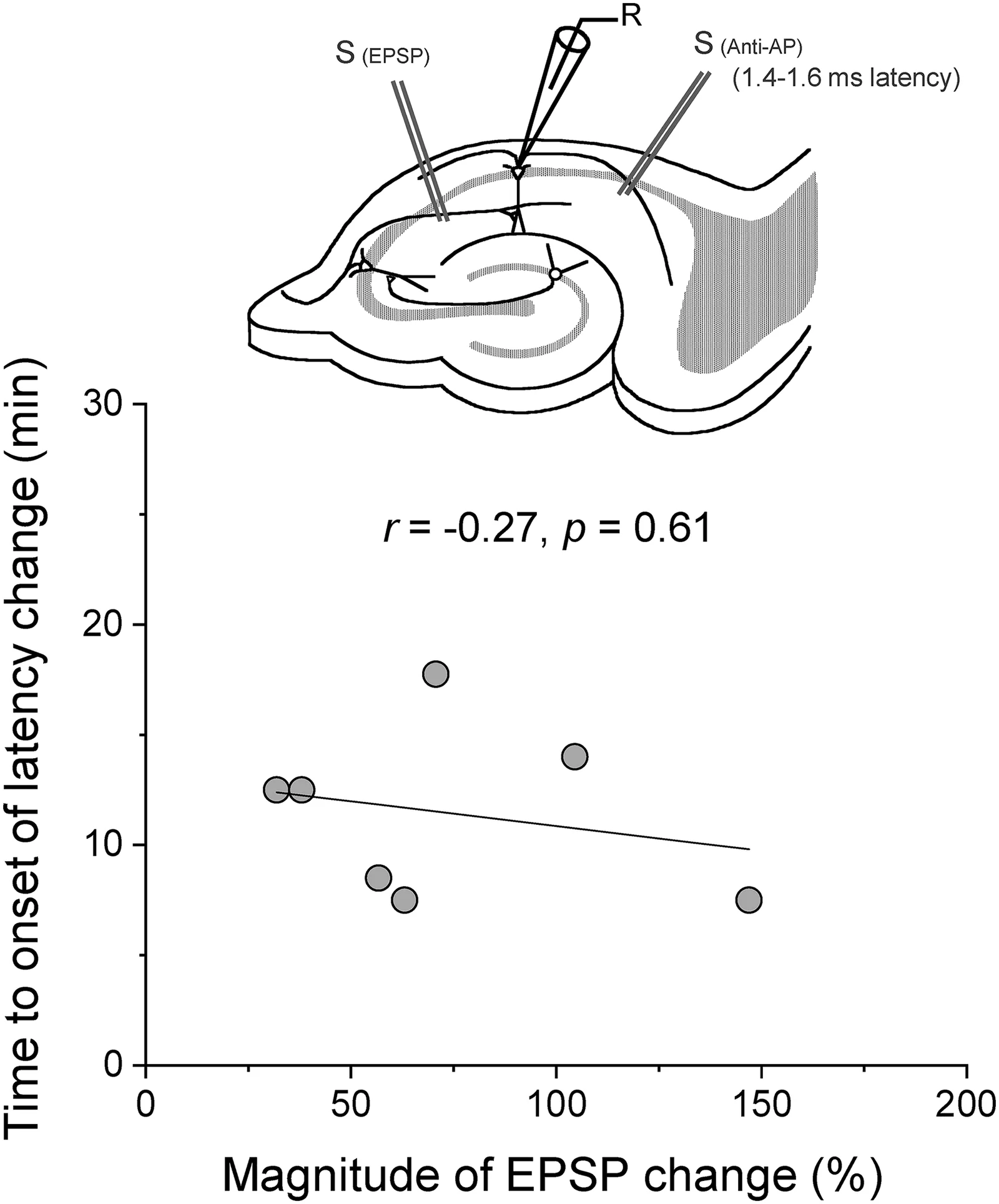
Synaptic potentiation magnitude does not determine the timing of axonal conduction changes at similar distances. ***A***, Schematic illustration of the recording configuration used for this analysis. Antidromic stimulation was applied at axonal sites selected to yield similar baseline antidromic action potential (Anti-AP) latencies (1.4–1.6 ms), thereby controlling for soma-to-stimulation site distance. ***B***, Scatter plot showing the relationship between synaptic potentiation magnitude and the onset time of Anti-AP latency changes in neurons with comparable baseline latencies (*n* = 7). Synaptic potentiation was quantified as the percentage change in the excitatory postsynaptic potential (EPSP) slope measured at 45–47 min after high-frequency stimulation, and latency onset time was defined as the first consistent reduction in Anti-AP latency. No significant correlation was observed.

Given that stronger LTP might be associated with enhanced neuronal activity and the increased production of molecular signals capable of driving axonal plasticity, we hypothesized that greater synaptic potentiation could lead to the earlier emergence of latency changes, even at more distal stimulation sites. Conversely, weaker LTP might result in delayed or absent changes in latency. To test this possibility, we examined whether the timing of latency change onset correlated with the magnitude of LTP in neurons with similar baseline latencies (1.4–1.6 ms). Specifically, latency onset time was plotted against the average EPSP slope measured at 45–47 min after HFS. However, there was no significant correlation between these two parameters (*n* = 7, *r* = −0.27, *p* = 0.61; Fig. 8), indicating that within this latency range, the timing of latency change onset was not determined directly by the extent of synaptic potentiation.

### Dual-site stimulation reveals spatial differences in the timing of axonal conduction modulation within single neurons

**Figure 9.**
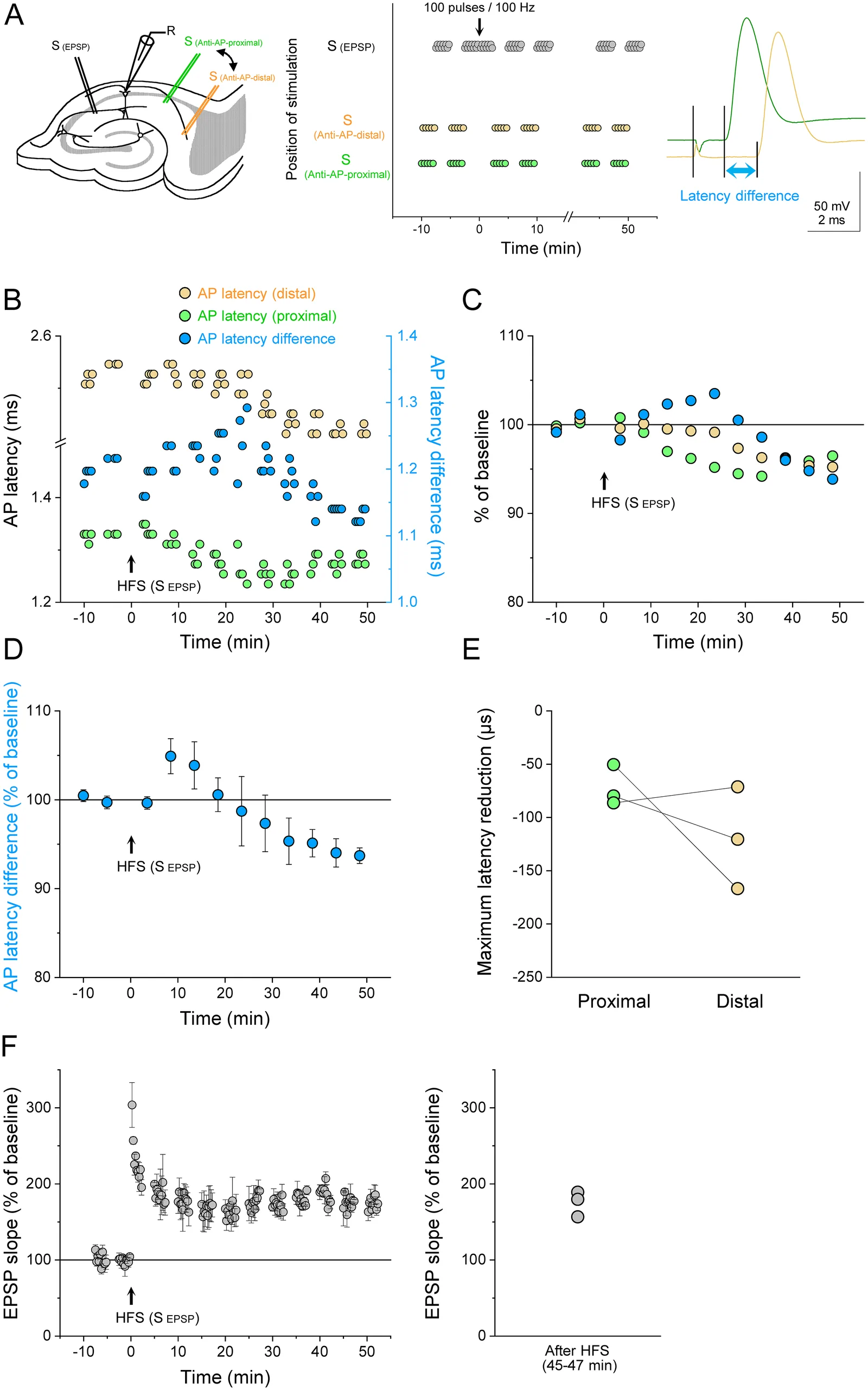
Dual-site antidromic stimulation reveals the spatially distinct timing of axonal conduction plasticity within individual neurons. ***A***, Dual-site antidromic stimulation paradigm. Left, Schematic showing the recording configuration, including the somatic recording electrode, the Schaffer collateral stimulation electrode for excitatory postsynaptic potential (EPSP) recording, and two separate axonal stimulation electrodes used to evoke antidromic action potentials (Anti-APs) with distinct latencies. The two axonal sites were stimulated in alternation. Middle, Stimulus and recording protocol illustrating the alternating acquisition of EPSPs and Anti-APs, with Anti-APs evoked sequentially from the short-latency and long-latency sites. Right, Representative Anti-AP waveforms evoked from the two axonal stimulation sites, illustrating the difference in latency and the definition of the latency difference used for analysis. ***B***, Representative recording showing the time course of absolute Anti-AP latencies evoked from the short- and long-latency sites, and the corresponding latency difference between both sites. ***C***, Same data as in ***B***, averaged across each set of five responses for each stimulation site and expressed as a percentage of baseline. Latency shortening emerged earlier at the short-latency site than at the long-latency site, resulting in a characteristic biphasic change in latency difference. ***D***, Group data showing the normalized time course of latency difference following HFS across neurons (*n* = 3). A transient increase in latency difference was observed during the early post-HFS period, followed by a gradual and persistent decrease. ***E***, Comparison of the maximal latency reduction observed at proximal and distal stimulation sites in individual neurons. Each pair of connected symbols represents measurements obtained from the same neuron. ***F***, Left, Time course of the EPSP slope recorded from the same neurons, confirming robust and sustained synaptic potentiation under the dual-site stimulation protocol. Right, Individual EPSP slope values measured at 45–47 min after HFS. Each symbol represents one neuron (*n* = 3).

To compare these temporal profiles directly, Anti-APs were evoked from two spatially separated axonal sites within single neurons. As in previous experiments, recordings were alternated between 10 EPSPs and 10 Anti-APs. In this protocol, the 10 Anti-APs consisted of five from the short-latency (proximal) site and five from the long-latency (distal) site, delivered in alternating order (Fig. 9*A*). The absolute latencies (in ms) for the short- and long-latency responses over time were plotted initially. The difference in latency between the two stimulation sites for each cycle was also calculated (Fig. 9*B*). On the basis of these data, the short-latency, long-latency, and latency difference values were then averaged over each set of five responses and expressed as a percentage of baseline, allowing direct comparisons of their respective time courses (Fig. 9*C*). As observed in earlier experiments, latency shortening emerged earlier at the short-latency site than at the long-latency site, reinforcing the idea that the timing of axonal conduction changes is influenced by the relative distance from the soma. Consistent with this temporal offset between stimulation sites, changes in the latency difference in a representative recording exhibited a characteristic time course. Following HFS, the latency difference initially increased, corresponding to the earlier emergence of latency shortening at the short-latency site, while the long-latency site remained unchanged. As latency shortening subsequently developed at the long-latency site, the difference in latency returned toward baseline and then continued to decrease below baseline, with this reduction persisting for the remainder of the recording period. We next examined whether this pattern was observed consistently across neurons. In group analysis of the difference in latency following dual-site stimulation (*n* = 3; Fig. 9*D*), a similar temporal profile emerged. Specifically, a transient increase in latency difference was observed during the early post-HFS period, followed by a gradual and sustained reduction that persisted throughout the remainder of the recording. Thus, at the population level, the difference in latency exhibited a biphasic time course, characterized by an early divergence and a later, persistent convergence of latency changes across both stimulation sites. We additionally compared the maximal latency reduction at the proximal and distal stimulation sites (Fig. 9*E*). Although the onset and peak timing of latency modulation differed between these sites, the magnitude of the maximal reduction of latency did not show a clear distance-dependent increase. In the same recording, robust LTP was also confirmed, as evidenced by a sustained increase in the EPSP slope following HFS (175.2 ± 9.6% of baseline, *n* = 3; Fig. 9*F*), indicating that the observed changes in latency difference occurred under conditions in which synaptic potentiation was induced. Importantly, the differential timing of latency changes between both stimulation sites within the same neuron argues against a purely somatic origin of the observed effects and instead supports the hypothesis that axonal conduction plasticity reflects local or spatially distributed modifications along an axon.

## Discussion

### Activity-dependent modulation of axonal conduction is temporally coupled to the stabilization of LTP

Recent studies have increasingly emphasized that axonal conduction, including myelinated and unmyelinated fibers, is not a static property but can be regulated dynamically by neuronal activity (Seidl, 2014; Fields, 2015). In this study, we demonstrate that the induction of synaptic plasticity is accompanied by a delayed and transient change in axonal conduction properties within the same neuron. By systematically comparing the time courses of synaptic potentiation and Anti-AP latency, our results reveal a close temporal association between axonal conduction plasticity and LTP stabilization.

Although the correlation between EPSP enhancement and antidromic latency reduction was strongest immediately after HFS (0–2 min), this early phase likely includes transient forms of synaptic enhancement such as posttetanic potentiation, rather than stable LTP alone. Posttetanic potentiation reflects short-lasting presynaptic enhancement immediately following HFS and is mechanistically distinct from the more persistent processes underlying stabilized LTP. Accordingly, the correlations observed during this early period should be interpreted cautiously, as they may reflect transient posttetanic synaptic activation rather than a stable relationship between synaptic and axonal plasticity. Importantly, significant correlations were also observed during the intermediate phase (25–27 min post-HFS), which temporally overlapped with the emergence and peak of antidromic latency reduction, as well as during the late phase (45–47 min), when synaptic potentiation had stabilized (Fig. 3). The persistence of this correlation during later phases indicates that axonal conduction changes are associated with consolidated synaptic modifications rather than transient poststimulation activity. The different analysis windows were selected to capture representative phases of two activity-dependent processes with distinct temporal profiles: stabilized synaptic potentiation and the maximal or near-maximal latency change. Therefore, the observed correlations should not be interpreted as indicating a direct relationship between EPSP slope and Anti-AP latency at two specific time points, but rather as reflecting the tendency for neurons exhibiting stronger synaptic potentiation to also exhibit larger latency modulation. The association between stabilized synaptic potentiation and latency modulation was further supported by recordings obtained under conditions that prevented stable LTP induction, such as intracellular Ca^2+^ buffering with BAPTA. Under these conditions, neither persistent synaptic potentiation nor latency reduction was observed, even when transient EPSP enhancement occurred immediately after HFS (Fig. 4). Together, these findings argue against a purely transient change in excitability and instead support the idea that axonal conduction plasticity is closely associated with enduring synaptic modifications rather than arising from transient poststimulation activity.

### Postsynaptic Ca^2+^-dependent signaling links synaptic and axonal plasticity

Although the suppression of LTP and antidromic latency shortening by intracellular BAPTA supports a close link between synaptic and axonal plasticity, the present experiments do not distinguish between Ca^2+^-dependent mechanisms intrinsic to LTP induction and those that may influence axonal conduction more directly. Discriminating between these possibilities would require approaches that selectively interfere with specific components of the LTP signaling cascade, such as Ca^2+^/calmodulin-dependent protein kinase II, a key mediator of NMDA receptor-dependent LTP induction (Lisman et al., 2012). Importantly, the present findings provide a framework for interpreting such future studies by demonstrating that axonal conduction plasticity emerges in conjunction with the successful induction and stabilization of synaptic potentiation. Although the changes in axonal latency were closely linked to LTP-related signaling pathways, their temporal persistence differed from that of synaptic potentiation. This distinction suggests that the mechanisms responsible for the induction of changes to axonal conduction may overlap with those underlying LTP, whereas the downstream processes governing maintenance and temporal stability are at least partially distinct. Such a dissociation is consistent with the broader principle that different forms of neuronal plasticity can share upstream signaling pathways while exhibiting different temporal dynamics and persistence.

### NMDA receptor signaling supports the stability of axonal conduction during intense synaptic activity

One of the most unexpected findings of the present study was that the blockade of NMDA receptors not only prevented LTP and axonal conduction facilitation but also revealed a small yet significant prolongation of antidromic latency when HFS was applied (Fig. 5). This result raises important questions regarding the role of NMDA receptor-dependent signaling in shaping axonal conduction properties during periods of intense synaptic activity. One possible interpretation is that the observed prolongation of latency does not reflect a direct suppressive effect of NMDA receptor blockade on axonal conduction per se, but rather a disruption of NMDA receptor-dependent processes that normally accompany LTP induction. Given the established role of NMDA receptors in activity-dependent synaptic integration and plasticity (Shouval et al., 2010), interference with these processes may indirectly compromise the mechanisms that maintain reliable axonal conduction following HFS. Alternatively, HFS itself may impose a transient burden on axonal conduction, which is normally counteracted by NMDA receptor-dependent signaling. The differential effects of intracellular BAPTA and NMDA receptor blockade suggest that antidromic latency modulation depends on multiple Ca^2+^-dependent signaling pathways rather than solely on NMDA receptor-mediated Ca^2+^ influx. Whereas intracellular Ca^2+^ buffering broadly suppressed latency changes, AP5 induced latency alterations with the opposite polarity (Fig. 5). Similar polarity-dependent regulation has been reported in synaptic plasticity, where interactions between distinct Ca^2+^ sources, including voltage-gated Ca^2+^ channels and IP_3_ receptor-dependent Ca^2+^ release, contribute to plasticity outcomes (Nishiyama et al., 2000). Such mechanisms may also contribute to the regulation of antidromic latency changes. Taken together, the AP5 results suggest that NMDA receptor-dependent processes contribute not only to synaptic potentiation but also to the maintenance of axonal conduction fidelity during periods of strong synaptic activation. The finding that AP5 alone had no effect on antidromic latency, whereas AP5 combined with HFS unmasked a conduction delay, points to a previously unappreciated interaction between synaptic and axonal plasticity mechanisms coordinated by NMDA receptor signaling.

### Axonal conduction plasticity is spatially organized along the axon

The present results demonstrate that the temporal profile of axonal conduction plasticity depends on the relative location of axonal stimulation, as estimated by baseline antidromic latency. The onset and peak of latency shortening occurred earlier at proximal sites, whereas comparable changes emerged with a systematic delay at more distal sites (Fig. 7). These findings indicate that axonal conduction plasticity follows a distance-dependent time course rather than being expressed uniformly along the axon. Baseline latency likely reflects a composite measure of electrotonic or physiological distance, integrating intrinsic conduction properties with the efficiency by which activity-dependent signals influence different axonal regions. The delayed emergence of latency changes at distal sites suggests that the underlying mechanisms involve processes that propagate or accumulate along the axon, rather than being engaged simultaneously throughout its length. Consistent with this view, although synaptic potentiation magnitude correlated with the extent of latency reduction, it did not predict the timing of latency change onset among neurons with similar baseline latencies (Fig. 8). This dissociation indicates that the temporal emergence of axonal conduction plasticity is not simply dictated by synaptic strength, but instead reflects spatial constraints on how plasticity-inducing signals spread or reach effective thresholds within the axon. Dual-site stimulation experiments further supported this conclusion: latency shortening emerged earlier but was transient at proximal sites, whereas it developed more slowly yet persisted at distal sites (Fig. 9). This pattern is inconsistent with a purely somatic or axon initial segment origin, which would be expected to produce similar temporal profiles at both sites, and instead indicates that axonal conduction plasticity is locally regulated along the axon. This within-neuron comparison places an important limitation on the underlying mechanisms by arguing against globally imposed changes in excitability. In addition, the magnitude of the maximal reduction of latency reduction did not show a clear distance-dependent increase between proximal and distal stimulation sites, despite the difference in onset and peak timing. This observation suggests that latency modulation is not expressed as a simple uniform change along the entire axonal path. In this context, latency difference analysis was used to assess within-cell changes in the relative timing between the antidromic responses evoked from both stimulation sites, thereby providing a measure of the spatial organization of latency modulation within individual neurons.

The activity-dependent regulation of intrinsic excitability mechanisms may nevertheless contribute to the observed latency changes. Recent studies have demonstrated that synaptic plasticity can be accompanied by structural and functional modifications of the axon initial segment, including the activity-dependent regulation of Na^+^ channel organization and geometry of the axon initial segment (Fréal et al., 2023; Sanz-Gálvez et al., 2025). Such mechanisms could influence antidromic spike initiation and propagation and may contribute to the latency changes observed here. In addition to mechanisms directly related to spike propagation along axons, other processes could contribute to the observed latency modulation. Short-term changes in somatodendritic conductance may influence action potential timing, and neuron–glia interactions have also been shown to regulate neuronal excitability and signaling dynamics (Deemyad et al., 2018). Therefore, although the present findings are consistent with the activity-dependent modulation of spike propagation, alternative mechanisms contributing to antidromic latency changes cannot be excluded completely. Changes in intrinsic excitability or Na^+^ channel availability could also influence Anti-AP latency (Colbert et al., 1997). However, sustained changes in resting membrane potential were not observed during the period in which latency shortening emerged, and any transient posttetanic membrane potential fluctuations were temporally distinct from the delayed latency changes analyzed here. Thus, posttetanic hyperpolarization alone is unlikely to account for the observed latency modulation. Since axonal conduction and action potential kinetics are temperature dependent, the quantitative magnitude of antidromic latency modulation may differ at physiological temperatures. This point should be considered when interpreting the effect size observed under the present recording conditions. However, the present analyses were based on within-cell changes measured under constant temperature conditions, and the observed latency modulation was associated with the induction of LTP and spatially organized temporal profiles, suggesting that it cannot be explained solely by the recording temperature.

Although axonal branching prevents definitive identification of the two stimulation sites as belonging to a single continuous segment, this limitation does not alter the central conclusion that axonal modifications are spatially organized. Together, these findings suggest that activity-dependent modulation of antidromic spike propagation exhibits a spatially and temporally organized structure that parallels key features of synaptic plasticity. While the magnitude of axonal conduction plasticity scales with synaptic potentiation, its timing and persistence are shaped by spatial factors intrinsic to the axon. This organization provides a mechanism by which neurons can fine-tune spike timing and signal propagation in a location-dependent manner, adding an additional layer of regulation to experience-dependent circuit modification.

### Mechanistic constraints on axonal conduction plasticity

Although the present study does not identify the specific ion channel subtypes involved or their precise subcellular localization, the results nevertheless provide important clues about the underlying mechanisms. The observed changes in antidromic latency imply regulated modifications in the determinants of spike propagation, including the threshold, reliability, and conduction speed of action potential transmission along the axon. These effects are most consistent with activity-dependent adjustments in the functional state of voltage-gated conductances that influence propagation speed, rather than nonspecific changes such as passive conduction shifts or activity-dependent fatigue.

The cellular locus of these mechanisms also remains to be determined. While the present data are consistent with cell-autonomous processes in the recorded neuron, they do not exclude contributions from periaxonal elements that may influence ion homeostasis or membrane properties. Thus, the present findings link synaptic potentiation to axonal conduction plasticity at a mechanistic level, while leaving open the potential involvement of glial interactions. A further limitation is that bicuculline was used as the methobromide salt, which has been reported to affect SK channels in addition to GABA_A_ receptors (Khawaled et al., 1999). In addition, because series resistance was not compensated during current-clamp recordings, some influence on the absolute measurement of fast voltage transients cannot be completely excluded. Nevertheless, bicuculline was present throughout baseline and post-HFS recordings, recording stability was monitored throughout the experiments, and the principal analyses were based on within-cell comparisons obtained under identical pharmacological and recording conditions.

From a functional perspective, the present findings highlight axonal conduction as a dynamic and plastic component of neuronal signaling. Synaptic plasticity regulates not only synaptic strength but also the timing of synaptic transmission. In cortical synapses, activity-dependent changes in synaptic efficacy are accompanied by corresponding changes in synaptic latency, such that potentiation shortens transmission delay whereas depression prolongs it (Boudkkazi et al., 2007). The present study focused specifically on HFS-induced synaptic potentiation, and it remains unclear whether other forms of synaptic plasticity induced by distinct temporal patterns of neuronal activity, such as spike timing-dependent plasticity or behavioral timescale synaptic plasticity, are accompanied by similar or distinct forms of axonal conduction modulation. Likewise, it remains unknown whether the synaptic depression induced by long-term depression protocols produces qualitatively different effects on antidromic latency and spike propagation dynamics. Future studies addressing multiple forms of synaptic plasticity will be required to determine how changes in synaptic efficacy are coordinated with the regulation of action potential timing. By showing that axonal conduction properties can be modified in association with synaptic potentiation—and in a spatially and temporally structured manner—this study suggests that neurons can fine-tune not only the strength of synaptic inputs but also the timing at which action potentials are delivered to downstream targets. Such coordinated modulation of synaptic efficacy and conduction timing may be particularly important for neural computations that rely on precise spike timing, including temporal summation and coincidence detection. Although the absolute changes in antidromic latency were relatively small, even submillisecond variations in spike timing may influence neuronal computation in temporally coordinated neural circuits. In particular, neuronal processing often depends on relative spike timing rather than absolute delay, such that small but systematic timing differences may influence synaptic integration and the temporal coordination of neuronal activity. Moreover, because the present measurements likely reflect timing changes occurring within only a limited portion of the axonal arbor, multiple local changes in spike propagation timing distributed across different axonal regions may collectively influence the timing of spike arrival and thereby exert broader effects on neuronal communication and network dynamics. This view is consistent with the concept that brain network operations are shaped by temporally structured patterns of neuronal activity, in which spike timing critically shapes circuit-level computation (Buzsáki and Mizuseki, 2014). In this context, the experience-dependent regulation of axonal conduction may provide an additional degree of freedom for shaping neural information processing beyond synaptic weight changes alone, consistent with theoretical frameworks emphasizing the role of spike timing in neural encoding (Rolls and Treves, 2011).

In summary, this study demonstrates that the induction of synaptic potentiation is accompanied by dynamic and spatially organized changes in axonal conduction within the same neuron. By simultaneously monitoring synaptic strength and Anti-AP propagation in hippocampal CA1 pyramidal neurons, we show that axonal conduction plasticity emerges with a delayed time course, is closely linked to NMDA receptor-dependent synaptic potentiation, and exhibits clear spatial dependence along the axon. Importantly, the timing and persistence of latency changes vary across axonal locations, arguing against a uniform or purely somatic origin and instead supporting locally regulated axonal modifications. Together, these findings reveal that axonal conduction is a dynamic and plastic component coordinated with synaptic plasticity, adding a previously underappreciated dimension to how experience shapes neuronal signaling and circuit function.

## Synthesis

Reviewing Editor: Viji Santhakumar, University of California Riverside Decisions are customarily a result of the Reviewing Editor and the peer reviewers coming together and discussing their recommendations until a consensus is reached. When revisions are invited, a fact-based synthesis statement explaining their decision and outlining what is needed to prepare a revision will be listed below. The following reviewer(s) agreed to reveal their identity: Rishikesh Narayanan. Note: If this manuscript was transferred from JNeurosci and a decision was made to accept the manuscript without peer review, a synthesis may not be available.

In this study, the authors examine the potential conjunctive plasticity of the canonical EPSPs (evoked by orthodromic stimulation) and novel action-potential latency (evoked by antidromic stimulation) in hippocampal pyramidal neurons in response to high-frequency stimulation. They observe a persistent long-term potentiation of synaptic strength accompanied by a transient change in AP latency. They demonstrate that the transient change in AP latency is calcium-dependent, with NMDARs acting as regulators of these transient changes. The overall question on conjunctive changes across different components is important for processing neuronal information, especially since biological plasticity is invariably multi-component. The idea of measuring axonal conduction velocity in conjunction with synaptic changes is noteworthy. However, there is concern that the mechanisms and functional relevance of these rather small and transient changes are overinterpreted. There are also several methodological details, data presentation, and analysis that need to be strengthened.

1. A major concern is the relatively small effect - a 100µs shift in latency that is transient. The physiological relevance of these limited changes needs to be addressed. For example, in Fig. 2B: Please provide a stick plot of the actual AP latency values before and after plasticity induction (even at the max-change values) for all recorded cells. Given the extremely small change that is reported, the authors are encouraged to evaluate the effect size (https://doi.org/10.1113/JP285575)

2. The transient nature of AP latency changes needs to be emphasized in the title, abstract, significance statement, and introduction.

3. Is this reduction in latency also observed when LTP is induced by STDP or BSTP protocol? In this regard, it would be interesting to determine how on latency is altered when LTD is induced.

4. Support for role of axonal conduction in observed plasticity: In the absence of direct evidence on AP latency being dependent on axonal conduction plasticity, those claims have to be toned down and should strictly be restricted to the discussion as one of several possibilities. The writing needs to improve, accounting for several possible interpretations. Alternate interpretations such as transient post-tetanic hyperpolarization of neurons (https://doi.org/10.3389/fnins.2022.881426; https://doi.org/10.1152/jn.00103.2012; https://doi.org/10.1113/jphysiol.1966.sp007918), change in sodium channel inactivation kinetics under hyperpolarization (see https://doi.org/10.1523/jneurosci.17-17-06512.1997); short term changes to somatic and/or dendritic ion channels, interactions between astrocytes and neurons impacting intrinsic properties of neurons (https://doi.org/10.1038/nn.4013; https://doi.org/10.1038/s41467-018-06338-3) need to be considered / discussed. Regarding the influence of sodium channel kinetics, can the authors plot RMP as a function of time and show that the transient hyperpolarization does not overlap with the transient change in AP latency that is being reported here? A set of experiments where RMP is strictly maintained throughout the experimental protocol will also address this possibility. Although Fig. 6 partly addresses this possibility, it does not rule out potential interactions between NMDARs and other calcium sources in altering mechanisms that could contribute to the observed phenomenon. Alternatively, especially with the HFS, the roles of astrocyte-neuron interactions or post-tetanic hyperpolarization cannot be ignored. Given these caveats, the evidence provided isn't sufficient to conclude that the observed changes are due to axonal conduction velocity changes. Therefore, please refine the language throughout to refer to findings as a change in antidromic AP latency, rather than axonal conduction plasticity. Experiments in Fig. 4 don't necessarily demonstrate that there is plasticity in axonal conduction velocity and could result from the alternate calcium-dependent mechanisms detailed above. This issue needs to be systematically addressed and language revised accordingly.

5. Relatedly, can authors reconcile the positive change in Figure 3B-C?: There are cells where the latency change is positive. This should be addressed.

6. Antidromic stimulation: The possibility of recruitment of orthodromic stimulation of synaptic receptors needs to be thoroughly evaluated using synaptic blocker experiments, especially for proximal vs. distal stimulation in the same cell. Antidromic stimulation is particularly tricky to establish, especially with synaptic receptors not blocked. What steps did the authors take to ensure that they are not activating synaptic receptors by the field stimulation? How deep was the stimulating electrode placed? What was the stimulus intensity required to elicit the antidromic response? Were there signatures of EPSPs that manifested in the recordings with antidromic stimulation? These are very important questions to address carefully, preferably with traces of antidromic recordings before and after plasticity. Please provide evidence for the ability to place antidromic stimulation electrodes at appropriate locations, without orthodromic stimulation being activated using synaptic blocker experiments (both excitatory and inhibitory synaptic receptors blocked; AMPA/NMDA/GABAA). Ideally, this should be conducted for both proximal and distal electrode positions in the same experiment and anti-AP waveform changes after receptor blockade should be reported. The differential recruitment of orthodromic synaptic activation could explain differences reported in Fig. 9, for instance.

7. Suggestions for Introduction: Please consider introducing the study in the context of the literature on activity-dependent axonal conduction velocity modulation through myelin remodeling. (https://doi.org/10.1126/science.1206998; https://doi.org/10.1126/science.1199139; https://doi.org/10.1016/j.cell.2013.12.038; https://doi.org/10.1016/j.conb.2020.10.022; https://doi.org/10.1038/s41582-022-00737-3; https://doi.org/10.1016/j.neuron.2019.10.013). Moreover, the authors should appropriately introduce the current view of multi-component plasticity, be it activity-dependent or neuromodulatory. The recognition that changes in astrocytes and oligodendrocytes, dendrites, intrinsic properties, extracellular matrix, etc., can contribute to plasticity is more mainstream (Kim and Linden, Neuron, 2008) and needs to be reflected in the introduction. Consider the suggested literature

https://doi.org/10.1038/nn1178

https://doi.org/10.1016/j.conb.2021.07.009

https://doi.org/10.1038/s41593-024-01597-4

https://doi.org/10.1016/j.neuron.2007.10.030

https://doi.org/10.1038/s41586-022-05146-6

https://doi.org/10.1038/s41586-024-08170-w

https://doi.org/10.1016/j.neuron.2012.09.010

https://doi.org/10.1523/JNEUROSCI.1201-23.2023

8. Please provide the rationale for the recording temperature at 30 degrees - this is less than physiological. Given the small change in latency observed here, the authors should discuss whether the phenomenon would still be present and valid at physiological temperatures.

9. Methods: The authors indicate that "EGTA in the internal solution was replaced with 10 mM BAPTA". The addition of 10 mM BAPTA would alter the osmolarity of the solution. If it is tetra potassium BAPTA, then the potassium would be 40 mM higher. How did the authors balance the osmolarity and the potassium concentration?

10. Methods: Please specify if the bicuculline was a methiodide or methbromide salt or if it was +bicuculine. Also mention the vehicle in which it was dissolved. Please justify the use of 1 µM bicuculline, which is not sufficient for full blockade of GABA_A receptors.

11. Methods: Please state the rejection criterion for series resistance values. Please mention if the series resistance was compensated for throughout the course of the experiments.

12. While presenting data, provide the actual numbers before and after for the important measurements in addition to the % change. Apart from providing Mean and SEM, please also provide the actual heterogeneities in the baseline measurements and how they change after plasticity, by using individual data points and connecting the pre and corresponding post data points in the plots. This will help readers appreciate the heterogeneity in plasticity across experiments.

13. Statistics: Please indicate if assumptions of normality in data were explicitly tested and how.

14. Line 132: -58 mV resting membrane potential, especially at 30 degrees, is a little too depolarized for CA1 pyramidal neurons from animals of this age.

15. The authors state that "This reduction was reversible, ...". The use of the term "reversal" here is not appropriate: Reversal typically means an active mechanism that was used to reverse a course of action. The term "transient" is more appropriate here. Please change throughout.

16. The authors state that they use two different time windows 50-52 min after HFS for EPSP slope and 23-25 min for mean Anti-AP latency. The use of two time windows needs to be adequately justified.

17. Fig. 9: While the authors have focused on the time of onset to latency change, could the authors evaluate the magnitude of change? Irrespective of the site of stimulation, the changes in AP latency are on the order of 100 µs. If the changes were in axonal conduction, shouldn't the effect size be larger for a longer propagation length than for a short propagation length of axonal propagation? How do the authors reconcile with the effect size being invariant to the distance of propagation? Moreover, as the latencies are with reference to two different stimulations, it is unclear if the analysis of latency-difference is appropriate here.

18. Experiments in Fig. 5 are reminiscent of synaptic plasticity studies demonstrating a graded dependence of polarity and strength of plasticity on calcium influx (https://doi.org/10.1038/35046067). Particularly, Fig. 4 shows that while transient changes to AP latency are calcium dependent, Fig. 5 shows that the plasticity is not just dependent on NMDAR calcium. The change in polarity of alterations suggests involvement of an additional calcium source (possibly VGCCs or IP3 This needs to be discussed.

19. Discussion: Please discuss PTP during the early period of measurement.

20. Discussion: If the plasticity AP latency were mechanistically coupled to LTP as the authors claim, why is it transient while LTP is persistent?

21. Discussion: Please discuss how the findings impact temporal processing of neuronal information. For instance, would LTP and LTD differentially affect AP latecy as has been shown with synaptic latency (Boudkkazi et al., Neuron 2007).

22.Discussion: Sodium channels in the axon are precisely regulated: synaptic and intrinsic plasticity are often observed in parallel. For instance, following LTD or LTP induction, the length of axon initial segment (AIS) is modified (see Fréal et al., Sci Adv 2023; Sanz-Galvez, Inglebert et al., BioRxiv 2025)? Perhaps, these modifications spill over the AIS and increase the excitability and conduction speed.

## Author Response

Point-by-point replies We thank the editor and reviewers for their careful reading of our manuscript and their thoughtful comments. Their suggestions are greatly appreciated and nearly all have been incorporated into the revised manuscript. Below you will find our point-by-point responses.

The changed and additional descriptions have been highlighted in yellow in the revised manuscript.

Comment 1. A major concern is the relatively small effect - a 100µs shift in latency that is transient. The physiological relevance of these limited changes needs to be addressed. For example, in Fig. 2B: Please provide a stick plot of the actual AP latency values before and after plasticity induction (even at the max-change values) for all recorded cells. Given the extremely small change that is reported, the authors are encouraged to evaluate the effect size (https://doi.org/10.1113/JP285575) To illustrate better the magnitude and consistency of the observed latency changes, we have revised Figure 2 to include paired plots of the actual antidromic latency values measured before and after HFS (23-25 min) for all recorded neurons. In addition, the corresponding changes in latency values are now provided to facilitate the evaluation of the magnitude of latency changes across individual recordings. These additions allow the direct visualization of the actual latency shifts, their variability, and the consistency of within-cell changes following the induction of plasticity. We have also clarified these points in the revised Results and Figure Legends. We have also expanded the Discussion to address the potential physiological relevance of small activity-dependent changes in axonal conduction timing, including the possibility that multiple local timing changes distributed along an axonal arbor may collectively influence spike timing and temporally coordinated neuronal processing.

Comment 2. The transient nature of AP latency changes needs to be emphasized in the title, abstract, significance statement, and introduction.

We agree that the transient nature of the observed antidromic latency changes should be emphasized more clearly throughout the manuscript. Accordingly, we have revised the title and terminology throughout the manuscript to reflect the transient and dynamically regulated nature of the observed latency modulation. These revisions were made to reflect the temporal properties of the observed conduction plasticity more accurately.

Comment 3. Is this reduction in latency also observed when LTP is induced by STDP or BSTP protocol? In this regard, it would be interesting to determine how on latency is altered when LTD is induced.

The present study specifically examined the axonal conduction changes associated with HFS-induced synaptic potentiation, and we agree that it will be important to determine whether other forms of activity-dependent plasticity induced by distinct temporal patterns of neuronal activity, such as spike timing-dependent plasticity or behavioral timescale synaptic plasticity, are accompanied by different forms of axonal conduction modulation. This point is also related to the reviewer's Comment 21 regarding the potential differential effects of distinct forms of synaptic plasticity on action potential timing. Therefore, we have expanded the Discussion to address these issues and to note that future studies will be required to determine whether LTD-inducing protocols produce qualitatively distinct effects on antidromic latency and axonal conduction dynamics.

Comment 4. Support for role of axonal conduction in observed plasticity: In the absence of direct evidence on AP latency being dependent on axonal conduction plasticity, those claims have to be toned down and should strictly be restricted to the discussion as one of several possibilities. The writing needs to improve, accounting for several possible interpretations. Alternate interpretations such as transient post-tetanic hyperpolarization of neurons (https://doi.org/10.3389/fnins.2022.881426; https://doi.org/10.1152/jn.00103.2012; https://doi.org/10.1113/jphysiol.1966.sp007918), change in sodium channel inactivation kinetics under hyperpolarization (see https://doi.org/10.1523/jneurosci.17-17-06512.1997); short term changes to somatic and/or dendritic ion channels, interactions between astrocytes and neurons impacting intrinsic properties of neurons (https://doi.org/10.1038/nn.4013; https://doi.org/10.1038/s41467-018-06338-3) need to be considered / discussed. Regarding the influence of sodium channel kinetics, can the authors plot RMP as a function of time and show that the transient hyperpolarization does not overlap with the transient change in AP latency that is being reported here? A set of experiments where RMP is strictly maintained throughout the experimental protocol will also address this possibility. Although Fig. 6 partly addresses this possibility, it does not rule out potential interactions between NMDARs and other calcium sources in altering mechanisms that could contribute to the observed phenomenon. Alternatively, especially with the HFS, the roles of astrocyte-neuron interactions or post-tetanic hyperpolarization cannot be ignored. Given these caveats, the evidence provided isn't sufficient to conclude that the observed changes are due to axonal conduction velocity changes. Therefore, please refine the language throughout to refer to findings as a change in antidromic AP latency, rather than axonal conduction plasticity. Experiments in Fig. 4 don't necessarily demonstrate that there is plasticity in axonal conduction velocity and could result from the alternate calcium-dependent mechanisms detailed above. This issue needs to be systematically addressed and language revised accordingly.

We have addressed these detailed concerns as follows.

First, regarding the interpretation of the observed latency changes as evidence of axonal conduction plasticity, we agree that the present study does not directly establish the precise biophysical mechanism underlying the reductions in Anti-AP latency. Therefore, we have revised the wording throughout the manuscript to avoid overinterpretation and to distinguish experimentally observed latency changes from definitive mechanistic conclusions regarding the activity-dependent modulation of spike propagation.

Second, regarding alternative interpretations, we have expanded the Discussion to acknowledge several additional mechanisms that could contribute to the observed latency changes, including changes in intrinsic excitability, Na+ channel regulation, somatic or dendritic conductance, and neuron-glia interactions. These possibilities are now discussed as important mechanistic considerations and limitations of the present study.

Third, regarding the possible contribution of post-tetanic hyperpolarization and Na+ channel inactivation effects, we did not observe sustained changes in resting membrane potential that paralleled the delayed time course of antidromic latency shortening. Any transient membrane potential fluctuations immediately following HFS were temporally distinct from the delayed latency changes analyzed in the present study, which emerged several minutes after HFS and persisted beyond the immediate post-tetanic period. We have clarified this point in the revised manuscript.

Fourth, regarding the interpretation of the BAPTA experiments, we agree that they do not by themselves establish the precise mechanism underlying the observed latency changes. Therefore, we have revised the text to clarify that the BAPTA results support a relationship between intracellular Ca2+-dependent signaling and antidromic latency modulation, while not excluding contributions from other Ca2+-dependent mechanisms.

Finally, the manuscript has been revised throughout to distinguish better the observed latency changes from their potential underlying mechanisms and to acknowledge important alternative interpretations and limitations.

Comment 5. Relatedly, can authors reconcile the positive change in Figure 3B-C? There are cells where the latency change is positive. This should be addressed.

Although the predominant effect of HFS was a reduction in antidromic latency, a small number of recordings exhibited little change or slight increases in latency. These observations are reflected in the variability of the correlation plots and are consistent with the heterogeneous magnitude of plasticity observed across individual neurons. We have clarified this point in the revised manuscript.

Comment 6. Antidromic stimulation: The possibility of recruitment of orthodromic stimulation of synaptic receptors needs to be thoroughly evaluated using synaptic blocker experiments, especially for proximal vs. distal stimulation in the same cell. Antidromic stimulation is particularly tricky to establish, especially with synaptic receptors not blocked. What steps did the authors take to ensure that they are not activating synaptic receptors by the field stimulation? How deep was the stimulating electrode placed? What was the stimulus intensity required to elicit the antidromic response? Were there signatures of EPSPs that manifested in the recordings with antidromic stimulation? These are very important questions to address carefully, preferably with traces of antidromic recordings before and after plasticity. Please provide evidence for the ability to place antidromic stimulation electrodes at appropriate locations, without orthodromic stimulation being activated using synaptic blocker experiments (both excitatory and inhibitory synaptic receptors blocked; AMPA/NMDA/GABAA). Ideally, this should be conducted for both proximal and distal electrode positions in the same experiment and anti-AP waveform changes after receptor blockade should be reported. The differential recruitment of orthodromic synaptic activation could explain differences reported in Fig. 9, for instance.

We agree that the antidromic stimulation experiments require the careful evaluation of possible synaptically mediated responses. In the present study, antidromic latency was defined as the interval between the stimulus artifact and the steep onset of the Anti-AP. Although alveus stimulation could evoke slower depolarizing components under some conditions, no obvious EPSP-like depolarization was observed immediately preceding the action potential onset used for latency measurements.

To address this concern directly, we performed additional experiments in the presence of AMPA, NMDA, and GABAA receptor antagonists. In proximal stimulation experiments, a slow depolarizing component following an Anti-AP was partially reduced by receptor blockade, indicating that synaptically mediated responses could contribute to the later portions of the recorded waveforms. However, the sharp onset of the Anti-AP and the measured latency were essentially unchanged. In distal stimulation experiments, receptor blockade had little effect on the waveforms and did not alter antidromic latency (Fig. 1F in the revision). Six of the seven recordings were obtained from paired stimulation sites within the same neuron, allowing a direct comparison of antidromic responses with different baseline latencies. Thus, although synaptic activation could contribute to later waveform components under some conditions, it did not measurably affect the latency measurements used in the present study.

These observations indicate that the latency measurements primarily reflect antidromic spike propagation and are minimally influenced by synaptic activation. Importantly, receptor blockade did not alter the characteristic antidromic waveform onset or latency measurements at either stimulation site, arguing against differential synaptic recruitment as the primary explanation for the site-dependent latency profiles shown in Figure 9.

We have added these control experiments to the revised manuscript and described the experimental procedures and results in the Methods, Results, and Figure Legends.

Comment 7. Suggestions for Introduction: Please consider introducing the study in the context of the literature on activity-dependent axonal conduction velocity modulation through myelin remodeling. (https://doi.org/10.1126/science.1206998; https://doi.org/10.1126/science.1199139; https://doi.org/10.1016/j.cell.2013.12.038; https://doi.org/10.1016/j.conb.2020.10.022; https://doi.org/10.1038/s41582-022-00737-3; https://doi.org/10.1016/j.neuron.2019.10.013). Moreover, the authors should appropriately introduce the current view of multi-component plasticity, be it activity-dependent or neuromodulatory. The recognition that changes in astrocytes and oligodendrocytes, dendrites, intrinsic properties, extracellular matrix, etc., can contribute to plasticity is more mainstream (Kim and Linden, Neuron, 2008) and needs to be reflected in the introduction. Consider the suggested literature https://doi.org/10.1038/nn1178 https://doi.org/10.1016/j.conb.2021.07.009 https://doi.org/10.1038/s41593-024-01597-4 https://doi.org/10.1016/j.neuron.2007.10.030 https://doi.org/10.1038/s41586-022-05146-6 https://doi.org/10.1038/s41586-024-08170-w https://doi.org/10.1016/j.neuron.2012.09.010 https://doi.org/10.1523/JNEUROSCI.1201-23.2023 We have revised the Introduction to place the present study within the broader context of the activity-dependent regulation of axonal conduction, including recent work on adaptive myelination and oligodendrocyte-mediated modulation of conduction properties. We have also expanded the Introduction to acknowledge the increasingly recognized view that neural plasticity arises from coordinated changes across multiple cellular and subcellular components, including synapses, intrinsic excitability mechanisms, glial cells, and axonal signaling pathways.

Comment 8. Please provide the rationale for the recording temperature at 30 degrees - this is less than physiological. Given the small change in latency observed here, the authors should discuss whether the phenomenon would still be present and valid at physiological temperatures.

We have clarified in the Methods that recordings were performed at 30{degree sign}C to maintain the stable long-duration whole-cell recordings required for the simultaneous assessment of synaptic potentiation and antidromic latency changes. We agree that action potential kinetics and axonal conduction are temperature dependent, and we have added a statement to the Discussion acknowledging this limitation. However, the present analyses were based on within-cell comparisons obtained under constant recording conditions, and the observed latency modulation was consistently associated with LTP induction and exhibited spatially organized temporal profiles. These findings indicate that the reported relationship between synaptic potentiation and antidromic latency modulation cannot be attributed simply to temperature-dependent variability. We have clarified this point in the revised manuscript.

Comment 9. Methods: The authors indicate that "EGTA in the internal solution was replaced with 10 mM BAPTA". The addition of 10 mM BAPTA would alter the osmolarity of the solution. If it is tetra potassium BAPTA, then the potassium would be 40 mM higher. How did the authors balance the osmolarity and the potassium concentration? To address this important concern, we have performed additional experiments in which the concentration of K-gluconate was reduced from 140 mM to 100 mM to compensate for the potassium introduced by BAPTA tetrapotassium salt. Under these adjusted conditions, HFS still failed to induce synaptic potentiation or antidromic latency shortening, yielding results comparable to those obtained with the original BAPTA-containing internal solution. These findings indicate that the suppression of synaptic potentiation and antidromic latency modulation was not attributable to the additional potassium introduced by the tetrapotassium form of BAPTA. We have revised the Methods to clarify the composition of the adjusted internal solution and added the corresponding control data to the Results and Figure 4.

Comment 10. Methods: Please specify if the bicuculline was a methiodide or methbromide salt or if it was +bicuculine. Also mention the vehicle in which it was dissolved. Please justify the use of 1 µM bicuculline, which is not sufficient for full blockade of GABAA receptors.

We have revised the Methods to specify that bicuculline was applied as bicuculline methobromide (methobromide salt of [+]-bicuculline) and was dissolved in distilled water before dilution in artificial cerebrospinal fluid. We have also clarified the rationale for using 1 µM bicuculline. This concentration was selected to partially reduce inhibitory tone while minimizing excessive network disinhibition and maintaining stable long-term recordings during simultaneous measurements of synaptic responses and Anti-APs. This approach is consistent with the concentration-dependent effects of bicuculline reported in hippocampal pyramidal neurons (Shirasaki et al., 1991).

Reference:

Shirasaki T, Klee MR, Nakaye T, Akaike N (1991) Differential blockade of bicuculline and strychnine on GABA- and glycine-induced responses in dissociated rat hippocampal pyramidal cells. Brain Res 561:77-83.

Comment 11. Methods: Please state the rejection criterion for series resistance values. Please mention if the series resistance was compensated for throughout the course of the experiments.

We have revised the Methods to clarify how recording stability was monitored during current-clamp recordings. Since the recordings were performed in current-clamp mode, access stability was assessed indirectly by applying small hyperpolarizing current steps and examining the resulting voltage responses, together with the continuous monitoring of action potential amplitude and waveforms throughout the experiments. Recordings were discontinued or excluded from the analysis if these measures changed by more than 25%, indicating the deterioration of recording quality. Series resistance was not compensated for during recordings.

Comment 12. While presenting data, provide the actual numbers before and after for the important measurements in addition to the % change. Apart from providing Mean and SEM, please also provide the actual heterogeneities in the baseline measurements and how they change after plasticity, by using individual data points and connecting the pre and corresponding post data points in the plots. This will help readers appreciate the heterogeneity in plasticity across experiments.

In line with the changes made in response to Comment 1, we revised Figure 2 to include paired plots of the actual Anti-AP latency values before HFS and during the period of maximal latency reduction after HFS, together with the corresponding changes in latency values for individual neurons. These plots show the baseline heterogeneity, within-cell paired changes, and magnitude of latency modulation in absolute units. We have described these additions in the Results and Figure Legends.

Comment 13. Statistics: Please indicate if assumptions of normality in data were explicitly tested and how.

Assumptions of normality were assessed using the Shapiro-Wilk test for datasets analyzed with parametric statistical methods. The results supported the use of parametric statistical analyses for the datasets examined. We have clarified this procedure in the revised Methods.

Comment 14. Line 132: -58 mV resting membrane potential, especially at 30 degrees, is a little too depolarized for CA1 pyramidal neurons from animals of this age.

We agree that resting membrane potential values in some recordings were relatively depolarized. However, the recordings included in the analysis were stable throughout the experiments, Anti-APs were elicited reliably, and analyses were primarily based on within-cell comparisons relative to each neuron's own baseline. In addition, no systematic drift in membrane potential was observed during the recording period. We have described these considerations in the revised Methods.

Comment 15. The authors state that "This reduction was reversible, ...". The use of the term "reversal" here is not appropriate: Reversal typically means an active mechanism that was used to reverse a course of action. The term "transient" is more appropriate here. Please change throughout.

We agree that the term "reversible" may imply the involvement of an active reversal process, whereas the present findings primarily demonstrate a transient reduction in antidromic latency. Therefore, we have replaced the term "reversible" with "transient" (or equivalent wording where appropriate) throughout the manuscript.

Comment 16. The authors state that they use two different time windows 50-52 min after HFS for EPSP slope and 23-25 min for mean Anti-AP latency. The use of two time windows needs to be adequately justified.

The different analysis windows were selected to reflect the characteristic temporal profiles of the synaptic potentiation and antidromic latency changes observed in the present experiments. The EPSP slope was quantified at 50-52 min after HFS to evaluate the stabilized phase of LTP, whereas antidromic latency was quantified at 23-25 min after HFS because latency shortening consistently reached its maximal or near-maximal level during this period. Thus, both time windows were intentionally chosen to capture representative phases of each form of plasticity rather than to compare identical post-HFS time points. We have clarified this rationale in the revised manuscript.

Comment 17. Fig. 9: While the authors have focused on the time of onset to latency change, could the authors evaluate the magnitude of change? Irrespective of the site of stimulation, the changes in AP latency are on the order of 100 µs. If the changes were in axonal conduction, shouldn't the effect size be larger for a longer propagation length than for a short propagation length of axonal propagation? How do the authors reconcile with the effect size being invariant to the distance of propagation? Moreover, as the latencies are with reference to two different stimulations, it is unclear if the analysis of latency-difference is appropriate here.

To evaluate the magnitude of latency modulation, we added a new panel (Fig. 9E) comparing the maximal latency reduction observed at the proximal and distal stimulation sites in individual neurons. Although the onset and peak timing of latency modulation differed between the stimulation sites, the magnitude of the maximal latency reduction did not show a clear distance-dependent increase. These findings suggest that the observed modulation is not expressed as a simple uniform change along the entire axonal path.

Regarding the latency-difference analysis, latency difference was used as a within-cell measure of relative timing between the antidromic responses evoked from the proximal and distal stimulation sites. Since both stimulation sites were examined within the same neuron, this analysis allowed us to assess spatial differences in the temporal evolution of latency modulation while minimizing variability arising from differences in absolute latency across cells. We have clarified these points in the revised Results, Discussion, and Figure Legends.

Comment 18. Experiments in Fig. 5 are reminiscent of synaptic plasticity studies demonstrating a graded dependence of polarity and strength of plasticity on calcium influx (https://doi.org/10.1038/35046067). Particularly, Fig. 4 shows that while transient changes to AP latency are calcium dependent, Fig. 5 shows that the plasticity is not just dependent on NMDAR calcium. The change in polarity of alterations suggests involvement of an additional calcium source (possibly VGCCs or IP3). This needs to be discussed.

We agree that the differential effects of intracellular Ca2+ buffering and NMDA receptor blockade suggest that antidromic latency modulation is not determined solely by NMDA receptor-mediated Ca2+ influx. Whereas intracellular BAPTA broadly suppressed latency changes, AP5 induced latency alterations with the opposite polarity, suggesting the involvement of additional Ca2+-dependent signaling pathways. Therefore, we have expanded the Discussion to consider the possibility that multiple Ca2+ sources, including voltage-gated Ca2+ channels and intracellular Ca2+ release pathways such as IP3 receptor-dependent signaling, contribute to the regulation of antidromic latency modulation.

Comment 19. Discussion: Please discuss PTP during the early period of measurement.

We have expanded the Discussion to address the possible contribution of post-tetanic potentiation during the early post-HFS period. In particular, we now clarify that the strong early correlation observed immediately after HFS may partly reflect transient synaptic enhancement mechanisms such as post-tetanic potentiation, which are mechanistically distinct from stabilized LTP. We further discuss that the antidromic latency changes emerged with a delayed time course and remained associated with the later stabilized phases of synaptic potentiation, supporting a relationship beyond transient post-tetanic enhancement alone.

Comment 20. Discussion: If the plasticity AP latency were mechanistically coupled to LTP as the authors claim, why is it transient while LTP is persistent? We agree that the transient nature of antidromic latency changes differs from the more persistent expression of LTP. Therefore, we have revised the Discussion to clarify that mechanistic coupling between synaptic potentiation and axonal conduction changes does not necessarily imply identical maintenance mechanisms. Rather, our findings suggest that these forms of plasticity may share upstream induction-related signaling pathways, while the downstream processes governing temporal persistence and stability are at least partially distinct. This distinction has now been explicitly discussed in the revised manuscript.

Comment 21. Discussion: Please discuss how the findings impact temporal processing of neuronal information. For instance, would LTP and LTD differentially affect AP latency as has been shown with synaptic latency (Boudkkazi et al., Neuron 2007).

We agree that the present findings raise the important possibility that different forms of synaptic plasticity may differentially regulate the timing of neuronal signaling. Previous studies have shown that changes in synaptic efficacy can be accompanied by corresponding changes in synaptic latency, with potentiation shortening and depression prolonging transmission delays (Boudkkazi et al., 2007). Although the present study focused specifically on HFS-induced synaptic potentiation, it is conceivable that other forms of synaptic plasticity, including LTD, may be associated with distinct forms of antidromic latency modulation. We have expanded the Discussion to address the potential implications of our findings for temporal processing and to discuss the possibility that the coordinated regulation of synaptic efficacy and spike timing may contribute to information processing in neural circuits.

Comment 22. Discussion: Sodium channels in the axon are precisely regulated: synaptic and intrinsic plasticity are often observed in parallel. For instance, following LTD or LTP induction, the length of axon initial segment (AIS) is modified (see Fréal et al., Sci Adv 2023; Sanz-Galvez, Inglebert et al., BioRxiv 2025)? Perhaps, these modifications spill over the AIS and increase the excitability and conduction speed.

We agree that the activity-dependent regulation of intrinsic excitability mechanisms, including plasticity of the axon initial segment (AIS), may contribute to the observed latency changes. Therefore, we have expanded the Discussion to incorporate recent studies demonstrating that synaptic plasticity can be accompanied by structural and functional modifications of the AIS, including the regulation of Na+ channel organization and AIS geometry (Fréal et al., 2023; Sanz-Galvez et al., 2025). Although AIS-associated mechanisms may contribute, the distinct temporal profiles observed at proximal and distal stimulation sites suggest that AIS-mediated changes alone are unlikely to account for the observed modulation of latency. Accordingly, we revised the manuscript to clarify that the observed latency modulation may involve multiple forms of activity-dependent excitability regulation, including AIS-associated mechanisms as well as additional spatially differentiated processes along the axon.
